# Pragmatic and contextualized methods selection for safety assessment of infant systemic exposure through human milk: the Milk4baby decision tree approach - a contribution from the concePTION project

**DOI:** 10.3389/fphar.2025.1602018

**Published:** 2025-08-05

**Authors:** Anaëlle Monfort, Julia Macente, Martje Van Neste, Miao-Chan Huang, Nina Nauwelaerts, Getahun Befirdu Abza, Ursula Winterfeld, Anne Smits, Karel Allegaert, Pieter Annaert, Monia Guidi, Alice Panchaud

**Affiliations:** ^1^ Service of Pharmacy, Lausanne University Hospital and University of Lausanne, Lausanne, Switzerland; ^2^ Drug Delivery and Disposition Lab, Department of Pharmaceutical and Pharmacological Sciences, KU Leuven, Leuven, Belgium; ^3^ Clinical Pharmacology and Pharmacotherapy, Department of Pharmaceutical and Pharmacological Sciences, KU Leuven, Leuven, Belgium; ^4^ Swiss Teratogen Information Service, Service of Clinical Pharmacology, Lausanne University Hospital and University of Lausanne, Lausanne, Switzerland; ^5^ Child and Youth Institute, KU Leuven, Leuven, Belgium; ^6^ Neonatal Intensive Care Unit, University Hospitals Leuven, Leuven, Belgium; ^7^ Department of Development and Regeneration, KU Leuven, Leuven, Belgium; ^8^ Department of Hospital Pharmacy, Erasmus MC, Rotterdam, Netherlands; ^9^ BioNotus, Niel, Belgium; ^10^ Centre for Research and Innovation in Clinical Pharmaceutical Sciences, University Hospital and University of Lausanne, Lausanne, Switzerland; ^11^ Institute of Pharmaceutical Sciences of Western Switzerland, University of Geneva and University of Lausanne, Lausanne, Switzerland; ^12^ Service of Clinical Pharmacology, Lausanne University Hospital and University of Lausanne, Lausanne, Switzerland; ^13^ Institute of Primary Healthcare (BIHAM), University of Bern, Bern, Switzerland

**Keywords:** lactation, infant exposure, medication safety, pharmacokinetics, clinical lactation studies, risk assessment, decision tree

## Abstract

More than 50% of women take at least one medication during lactation. However, 54% of drugs in the LactMed database lack lactation safety data, and only 2% have robust evidence. This highlights a significant gap in guidance for designing pharmacokinetic and safety studies characterizing infant safety following medication exposure during lactation, despite FDA guidelines recommending clinical lactation studies. Additional guidance is needed to select the most suitable study design for these studies. To address this, we identified key medication-related characteristics essential for designing lactation studies that assess infant safety following systemic exposure during lactation. This allowed us to develop a decision tree, named Milk4baby, to guide researchers in selecting the most appropriate methodological approach for each medication. Milk4baby was designed by reviewing the literature and iterative discussions with an interdisciplinary panel of experts in clinical pharmacology, lactation, and pharmacometrics on factors influencing the selection of the methodological approach and design of a lactation study. The decision tree first considers the prevalence of medication utilization in women of childbearing age. Next, the medication’s safety profile in infants aged 0–2 years must be assessed using available safety data from infants, adults, and/or animals. Finally, the expected infant systemic exposure level is evaluated based on medication’s oral bioavailability, transfer into human milk, risk of accumulation, and utilization patterns. After completing these steps, the decision tree recommends the most suitable methodological approach including case reports/case studies, population pharmacokinetic (popPK) modeling, physiologically based pharmacokinetic (PBPK) modeling and simulations, or pharmacoepidemiologic studies. Verification of the decision tree on 50 randomly selected medications from the LactMed and Le CRAT databases revealed that PBPK and case reports were the most appropriate approaches in 29 cases, primarily due to low prevalence of medication utilization. Designing popPK, PBPK, or pharmacoepidemiologic studies can be time-consuming and resource-intensive, while poorly designed case reports/case studies may yield limited or misleading information. Therefore, Milk4baby aims to help researchers enhance the efficiency and accuracy of determining infant safety following systemic exposure during lactation by choosing the most suitable strategy for lactation studies, ultimately supporting better-informed decisions for lactating women and their healthcare providers.

## 1 Introduction

In 2023, the World Health Organization (WHO) reported that 48% of women breastfeed, a figure approaching the World Health Assembly’s 2025 target of 50%. This marks a 10% increase over the past decade, reflecting changes in lactation practices and growing interest in the health of lactating mothers (World Health Organization, 2023). However, the utilization of medications during lactation is also prevalent with up to 90% of lactating women reporting medication utilization in the months following childbirth (Saha et al., 2015). The rise in medication utilization during this period is largely driven by the need to treat postpartum common health issues, as well as the emergence of new therapies for chronic conditions and the trend of later maternal age at childbirth. The most frequently utilized classes of medications during this period include those targeting the nervous system, the genitourinary and endocrine systems, the cardiovascular system, the musculoskeletal system, and systemic anti-infective agents (Lutz et al., 2020). While the majority of medications are utilized for short-term periods, typically lasting 7 days, some may be administered for a duration exceeding 60 days, especially in the case of underlying chronic diseases. Currently, mothers are too often discouraged from starting or continuing lactation when they need medication or choose to discontinue the medication or postpone its initiation during the lactating period, regardless of the risk associated with the untreated disease for both them and their infant. Additionally, women are prescribed medications “off label” while lactating, without understanding if it is related to potential risks for the infant. With the high prevalence of utilization observed, it seems crucial to collect safety information with a good level of evidence for medications during breastfeeding, as most of them transfer into human milk. Furthermore, the potential risks of not lactating should be carefully weighed when evaluating the overall risks and benefits of medication utilization during lactation (Centers for Disease Control and Prevention, 2011b).

Unfortunately, the number of studies evaluating the safety of medications during breastfeeding remains limited. This scarcity of data arises from the exclusion of pregnant and lactating women in clinical trials during drug development (Sportiello and Capuano, 2023). However, efforts are ongoing to change both the perception and practices. Regulatory agencies are issuing new recommendations aimed at increasing the inclusion of these populations in clinical studies, notably through initiative like the task force on Research Specific to Pregnant Women and Lactating Women (PRGLAC). This task force was established to identify and address the gap in knowledge and research on safe and effective therapies for pregnant and lactating women (Byrne et al., 2020). In 2019, the U.S. Food and Drug Administration (FDA) drafted recommendations for sponsors conducting clinical lactation studies (Food and Drug Administration, 2019). These recommendations have led to an increasing number of Post Marketing Requirements (PMR) related to lactation. For instance, in 2019 and 2020, 6 and 3 PRMs, respectively, were issued for medication utilized during lactation, compared to only 3 between 2007 and 2016 (Krastein et al., 2023). Despite these efforts, the number of medications with no or limited safety data on breastfeeding remains concerning. In 2018, 54% of the 1,408 drugs listed in the National Library of Medicine’s comprehensive database on drugs and lactation, LactMed, had no breastfeeding-related safety data, and only 2% had sound safety evidence (Byrne and Spong, 2019). Not only is the quantity of available data worrying, but so is its quality. Methodological bias and high inter-individual variability in data often prevents the assessment of safety risk in breastfed infants. Most available data come from case reports, which, while often useful, can be misleading. An example of these quality issues is illustrated by a case report on codeine which attributed the death of an infant to maternal codeine utilization during breastfeeding (Koren et al., 2006). Years later, this case was retracted by two journals, and codeine was demonstrated to be no more dangerous than other opioids (Zipursky and Juurlink, 2020; Tsuyuki and Pimlott, 2021). To avoid such inconsistencies and variability in data, Anderson et al. proposed specific information that should be reported in case reports to improve the usefulness and quality of case reports (Anderson, 2022). Similar standards should be applied to all types of lactation studies.

Evaluating medication safety during breastfeeding requires determining both the potential adverse effects on breastfed infants and the prevalence of these effects, ideally weighted to the expected incidence. Such data can only be obtained through pharmacoepidemiologic safety studies, which, although very informative, can take years to conduct and may not always be feasible (Jordan et al., 2022). As an alternative, infant systemic exposure can be assessed by studying the pharmacokinetics of medication excretion into human milk and the associated infant systemic exposure. Ideally, this would involve measuring medication concentrations in the infant’s blood to accurately describe the exposure. However, this method is rarely used due to the invasive nature of blood collection, which often hinders parental consent. As a result, the most common, straightforward, and non-invasive approach to assessing infant systemic exposure involves measuring medication concentrations in human milk and calculating the daily infant dose (DID) and the relative infant dose (RID) (Anderson, 2018). These markers can help estimate medication levels in infants, but they often rely on the assumption that the pharmacokinetics in infants is similar to that of adults. However, this assumption is not completely accurate, particularly for neonates whose medication absorption, distribution, metabolism, and excretion can differ significantly from adults (Milsap and Jusko, 1994). These differences arise from major physiological distinctions between adults and infants. The situation gets even more complex when considering that the physiology of children changes significantly with age. Oral absorption in infants differs from that in adults due to variations in gastric pH, gastric emptying, intestinal transit time, and the ontogeny of first-pass metabolism. Medication distribution is influenced by differences in membrane permeability, unbound fraction, and body water content. Metabolism is affected by the immaturity of metabolic enzymes, and excretion is altered due to the immaturity of glomerular filtration and renal tubular secretion (Fernandez et al., 2011). RID remains the most reliable marker for assessing infant systemic exposure during breastfeeding, but it should account for the pharmacokinetic differences between infants and adults when estimating infant medication levels.

Various methodological approaches exist to assess infant systemic exposure during lactation. The most commonly used are case reports and case series which are generally designed to document unusual, novel, or complex medical observations (Cardoso et al., 2023a). In the area of medication safety in lactation, these methods are typically employed to report the presence or absence of adverse events in breastfed infants who are exposed to a certain medication through lactation. These reports are often supplemented with medication concentration measurements from one or more human milk samples. They frequently provide the first pharmacokinetic information on medication excretion into human milk. From these measurements, the milk-to-plasma (M/P) ratio can be calculated using either a single time-point or the 24-h area under the curve (AUC) of concentrations in human milk and maternal plasma (Anderson, 2018). Case reports and case series offer several advantages: they are opportunistic, low-cost, do not require patient recruitment processes, have a short follow-up period and are easy to share within the scientific community. However, they provide limited evidence, as they cannot establish causality between medication exposure (i.e., medication in human milk) and outcome (e.g., side effects in the breastfed infant). They also have a poor representativeness due to the lack of population-level estimates, suffer from variability in medication concentrations associated with highly variable sampling times, making results difficult to interpret, and their findings cannot be generalized. Additionally, they often lack prospectively collected information and are prone to publications bias. Traditional pharmacokinetic studies, which use intensive blood and milk sampling from individuals, are another possible approach to assess infant medication exposure through human milk. This method is relatively straightforward, requires a small sample size, and involves simple calculations. However, it fails to quantify inter-individual variability and to identify its underlying sources, and requires multiple sampling from each breastfeeding mother, which can be burdensome and considered unethical. In recent decades, new methodological approaches have emerged for evaluating infant systemic exposure to medications during lactation. These methods include population pharmacokinetic (popPK) and physiologically based pharmacokinetic (PBPK) modeling. Although still underutilized in this field, these methods have shown promise in studying medication transfer into human milk (Delaney et al., 2018; Weisskopf et al., 2020; Panchaud et al., 2011; Monfort et al., 2024; Melander et al., 2025). PopPK modeling, a top-down approach, uses *in vivo* human data, i.e., milk and plasma concentrations, from multiple individuals to describe the pharmacokinetic profile of a population. This method also helps identify demographic, environmental, or genetic factors contributing to inter-individual variability. It requires only a few samples per patient, which can be retrieved from different heterogenous studies, and can predict medication exposure through simulations. However, popPK analysis requires a larger number of clinical data, which can be a limitation in clinical lactation studies. Additionally, the reliability and predictive performance of popPK models depend heavily on the availability and quality of the collected data, emphasizing the need for well-designed lactation studies. PBPK, a mechanistic approach, uses population-specific physiological parameters and medicine specific properties derived from *in silico*, *in vitro* and *in vivo* animal experiments to predict (simulate) the pharmacokinetic profile in individuals. By incorporating variability in these parameters, it can characterize the pharmacokinetics in a population. Limited clinical data can then be used to assess the predictive performance of the model. The main advantage of PBPK modelling and simulations is that it does not strictly require clinical data, except for model verification, making it useful for medications where recruiting lactating women is challenging. However, PBPK is a complex methodological approach that remains underdeveloped, and its predictive performance relies heavily on the availability and quality of input data (Cardoso et al., 2023a). In the context of lactation studies, there is a need for a better understanding of lactation anatomy, physiology, human milk composition, population variability, and functional changes over the postpartum period (Van Neste et al., 2023).

Each of the methodological approaches described above has its advantages and limitations and should be selected after assessing the medication’s context of utilization and safety profile. This article, therefore, proposes a decision tree approach to determine the most pragmatic and suitable methodological approach for safety assessment in infants after systemic exposure to medications during lactation in the post-marketing phase, depending on the medication studied. This decision tree, named Milk4baby, aims to provide a roadmap to inform the decisions of regulators, manufacturers, researchers and healthcare providers in the design and/or interpretation of future milk studies. Additionally, we intend to identify critical parameters to consider when designing clinical lactation studies besides the methodological approach.

## 2 Development of the Milk4baby decision tree

### 2.1 Key factors for the Milk4baby decision tree development

**FIGURE 1:**
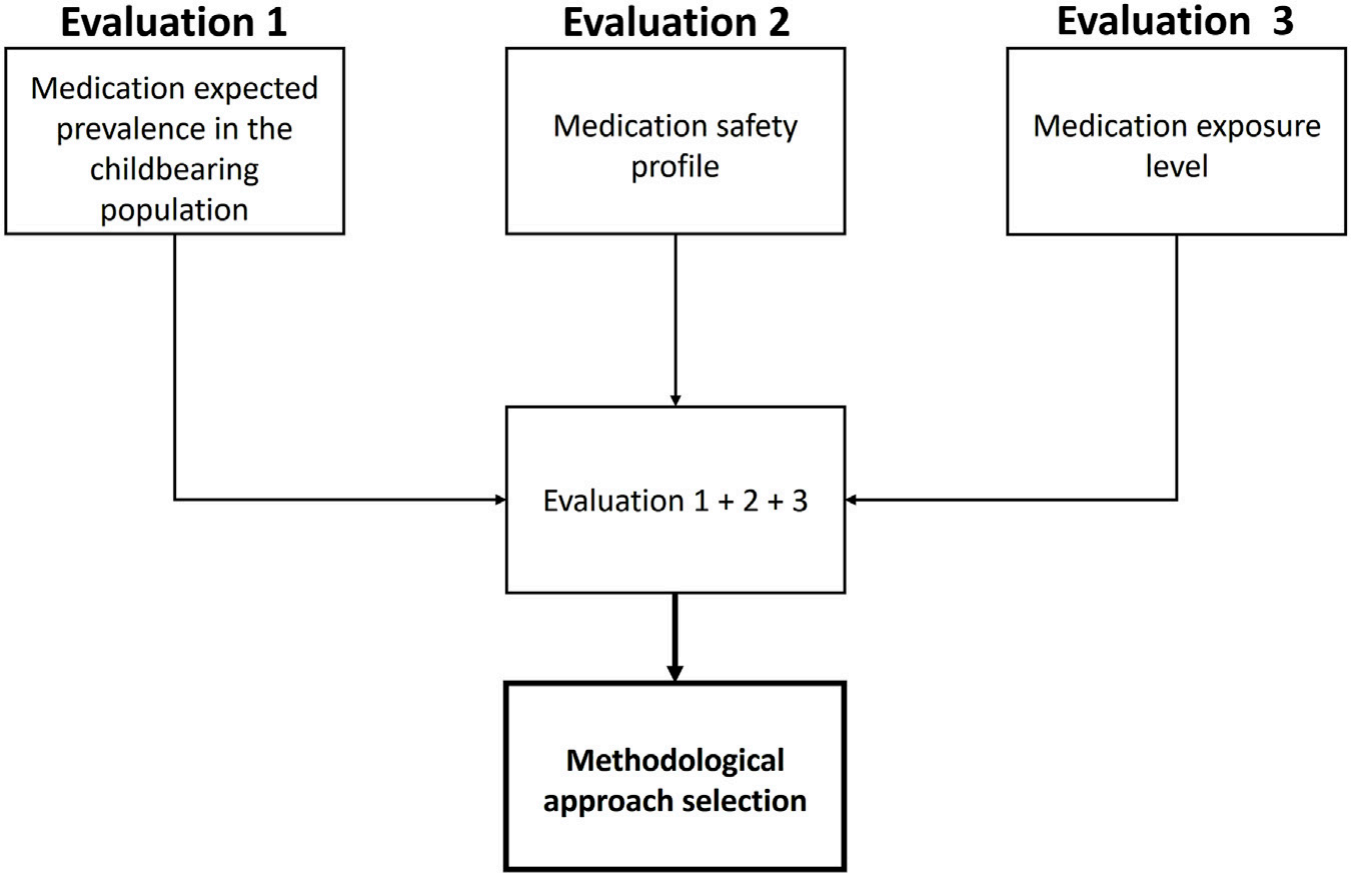
Overview of the Milk4baby decision tree.

We first identified the main factors that will influence the selection of the methodological approach and design of a lactation study, to ensure a feasible and cost-effective choice. These key factors were selected by an interdisciplinary panel of experts (i.e., in pharmacology, lactation, neonatology, popPK, and PBPK) and a literature review assessing previously used factors to evaluate infant safety profile after systemic exposure to medications during lactation, as well as scales to assess the impact of each factor. This iterative process led to the identification of three key factors that seemed to weight significantly in the selection of a pragmatic and contextualized methodological approach: the expected prevalence of medication utilization in the childbearing population, the medication safety profile, and the expected medication level of exposure in breastfed infants (Figure 1).

Another important aspect to consider is the ontogenetic continuum of the exposed population of interest. The WHO and the American Academy of Pediatrics (AAP) recommend exclusive breastfeeding for the first 6 months, continuing partially for up to 2 years with complementary food (World Health Organization, 2024; McGuire, 2011; European Commission Directorate Public Health and Risk Assessment, 2008). This period includes neonates (≤28 days) and infants (between 28 and 2 years). When assessing medication level of exposure, it is essential to be aware that pharmacokinetics can differ significantly between children and adults, notably during the early years of life due to ontogenetic and physiological differences (Batchelor and Marriott, 2015). Furthermore, pharmacokinetics can vary substantially within our population of interest, particularly between neonates and older infants, making the infant’s age a critical factor to consider. These differences include, for example, reduced intestinal transit time, lower gastric pH, increased levels of fat mass and water, decreased plasma protein concentration and a larger relative size of the liver and kidneys, all of which can modify the medication’s absorption, distribution, metabolism, and excretion. Similar considerations can be made for medication safety profile (Milsap and Jusko, 1994). Consequently, each key factor should be evaluated with respect to the specific age group being studied, and the lactation study should be designed accordingly. If no specific age group is targeted, particular attention should be given to infants at the highest risk of exposure or adverse effects, namely, the youngest and exclusively breastfed infant, typically those under 6 months of age.

#### 2.1.1 Expected prevalence of medication utilization in the childbearing population

The choice of the methodological approach to assess infant safety profile after systemic exposure to maternal medications during lactation relies heavily on the number of women taking the medication during the childbearing period. Indeed, some of the available assessment approaches require a significant number of participants and biological samples, which may not be achievable for all medications. Therefore, the first step in selecting the methodological approach for determining the safety profile of infants after systemic exposure during lactation is to assess the expected prevalence of medication utilization in lactating women. Women of childbearing age appear to be the most representative of the prevalence of medication utilization during lactation as restrictions on utilization in pregnant and lactating women due to the lack of safety data for these two populations is likely. Nevertheless, one should be aware that medication prevalence in the childbearing population is often derived from the pregnancy population, which is more extensively studied. Although not entirely representative of the lactating population, it is likely that a medication utilized during pregnancy might also be utilized during lactation (Anderson, 2018). On the other hand, if a medication is not utilized during pregnancy due to a teratogenic profile, this does not necessarily mean that this medication will not be utilized during lactation, as it might actually be considered safe during this period.

We could also argue that data on medication utilization, specifically in the lactating population should be considered. However, based on our literature review, these data are rarely available (Saha et al., 2015; Jordan et al., 2022; Ceulemans et al., 2022; Soliman et al., 2024). Due to the increase in lactation rates over the past decade (World Health Organization, 2023), the childbearing population seems to currently better represent the lactating population. If the prevalence of specific medications in the childbearing population is not well-documented in the literature, the evaluation should rely on the prevalence of medication utilization in the general population and be weighted towards expectations in lactating women. For instance, a medication frequently utilized in the general population but prescribed primarily for an indication in men would be classified as rare among lactating women.

For the purpose of the development of the Milk4baby decision tree, we have considered it reasonable to categorize this factor into 3 groups, either low, intermediate, or high prevalence of medication utilization in the childbearing population or in the general population, if suitable. Medications with low prevalence of utilization in the childbearing population include orphan medications utilized for rare diseases, defined as fewer than 50 cases per 100,000 persons in Europe (0.05%) and 1 case per 100,000 persons in the US (0.001%), accidental utilizations, some off label utilizations, or treatments not intended for women or utilized later in life (Danese and Lippi, 2018). Examples of such medications include lanreotide, utilized to treat acromegaly, a rare disease, and tamsulosin, approved for men only but utilized off-label in women to treat kidney stones. Intermediate prevalence encompasses medications utilized to treat conditions that are uncommon in lactating women or common conditions where the medication is not typically a first-line treatment (i.e., between 0.05% and 0.1% in the childbearing population). This category may include treatments for fertility issues or ectopic pregnancies. For instance, the prevalence of ectopic pregnancy following a first intrauterine pregnancy is 0.6%, and the prevalence of having a second child in the first 2 years after a first pregnancy is 23% (Chouinard et al., 2019). Methotrexate is the first-line treatment to treat ectopic pregnancies but is also contraindicated during pregnancy due to its teratogenic profile. Another example is fluvoxamine, utilized to treat depression, a common condition in lactating women, but not as a first-line treatment. Finally, high prevalence includes medications utilized to treat common conditions in lactating women (>0.1% of utilization in the childbearing population). This category encompasses oral contraceptives (>8%), antidepressants (>7%), antibiotics (>2%), nonsteroidal anti-inflammatory agents (<1%), and antihypertensive medications (around 1%), for example, (Palmsten et al., 2023; Molenaar et al., 2020; Schirm et al., 2004). These medications are commonly prescribed during lactation.

#### 2.1.2 Medication safety profile

The choice of the methodological approach to assess the infant safety profile following systemic exposure to maternal medications during lactation will also depend on the toxicity profile of the studied medication. The options may vary from a no clinical sample related approach to a shorter evaluation, enabled by a well-documented low toxicity profile in pediatrics (Centers for Disease Control and Prevention, 2011a). The best indicator of a medication’s safety in breastfed infants is its safety when administered directly to infants of the same age (i.e., under 2 years old). Safety can be assessed by examining the type, severity, and frequency of adverse effects in infants. For the purpose of the development of the Milk4baby decision tree, we have considered it reasonable to categorize this factor into 3 groups, safe, moderately safe and unsafe, based on literature review.

A medication is deemed safe if no or only Type A adverse effects, i.e., predictable and dose-dependent, causing only trivial or mild symptoms such as a rash or gastrointestinal issues, are observed. These effects typically do not require dosage adjustments or intervention. A medication is categorized as moderately safe if the symptoms result in some impairment but are not life-threatening, are uncomfortable and interfere with activities, potentially requiring dosage adjustments, or minimal, local or noninvasive intervention. Finally, a medication is considered unsafe if adverse effects are Type B, unpredictable, severe, or life-threatening, mandating withdrawal of the medication (Edwards and Aronson, 2000; Aronson and Ferner, 2005).

Safety data may not be available for the specific subgroup of children under 2 years of age. When data are available for this subgroup, priority should be given to infants at the highest risk, specifically those who are exclusively breastfed and less physiologically mature. In order of priority, this includes infants below 2 months of age, followed by those below 6 months, and finally, those under 2-years old. If no data on medication safety are available for the infant population, then we should first refer to safety data in children under 12 years of age and then in the general population, using the same classification framework. Despite the safety profile of the medication in older children or in adults, caution is warranted when simply extrapolating to infants due to ontogenetic, physiological and pharmacokinetic differences presented earlier (Milsap and Jusko, 1994). Indeed, if a medication is unsafe for older children or adults, it is likely unsafe for infants. However, the opposite is not necessarily true. Finally, if the safety of the medication is unknown in the general population, data on medication safety might be available in animals. Toxicity is considered established in animals, if preclinical toxicology studies have been conducted in at least two mammalian species, including one non-rodent species (European Medicine Agency, 2009). Although animal study may provide pertinent information, the medication should be classified as high risk for the breastfed infant, unless human data exist. In such cases, the design of the lactation study should also be adapted to ensure that the medication’s safety profile is thoroughly evaluated in breastfed infants.

A conservative approach should always be used when assessing the medication’s safety profile. Thus, in cases of uncertainty, the highest risk category should be selected.

#### 2.1.3 Medication exposure level

##### 2.1.3.1 Mother exposure

To evaluate the expected level of medication exposure in infants during lactation, we must reconstitute the medication from its administration to the mother to the plasma concentrations observed in the infant. The first step involves assessing the medication’s systemic absorption in the mother. Absorption is the process by which the medication reaches systemic circulation and is a key factor in determining the medication’s bioavailability (Paul et al., 2019), which is also influenced by presystemic (first-pass) metabolism in the case of oral administration. Bioavailability is, therefore, a crucial parameter for assessing infant systemic exposure to the medication. Medication bioavailability is significantly dependent on the mode of administration. For instance, bioavailability in systemic circulation is generally limited after topical administration, thereby restricting the medication’s passage into human milk, while intravenous injection maximizes medication bioavailability, enabling a higher level of transfer into human milk. Consequently, the presence or absence of systemic bioavailability, as well as the medication’s ability to reach therapeutic concentrations in the mother, must be assessed based on the mode of administration of the medication. For prodrugs like clopidogrel, the active metabolites should be considered when evaluating the attainment of therapeutic concentrations. Special consideration should also be attributed to topical medications applied to the nipples, as they may not enter systemic circulation but can still be transferred to human milk or ingested directly by the breastfed infant. Once the presence or absence of systemic bioavailability in the mother is determined, the next step is to estimate the quantity of the medication that will transfer into human milk. To reach human milk, medications must first be absorbed in the systemic circulation of the mother. Therefore, if a medication does not enter the systemic circulation, it cannot be transferred to the infant through human milk, and the level of exposure will be zero. Since this study aims to guide the design of clinical lactation studies, we assume no existing clinical data on medication transfer into human milk or limited data that require better characterization. Thus, this transfer should be estimated using the medication’s physicochemical properties (e.g., molecular weight, logP, pKa, protein binding), pharmacokinetic characteristics (e.g., half-life, volume of distribution, metabolites) and existing clinical data, if available (Cardoso et al., 2023b).

##### 2.1.3.2 Infant exposure

Next, one should assess whether the breastfed infant will exhibit systemic exposure to the medication after oral “administration”. Indeed, only oral bioavailability should be considered, as breastfed infants are exclusively exposed to medication through oral intake. For the purpose of our study, three arbitrary bioavailability classes were considered, based on pharmacokinetic understanding and literature. Low bioavailability was defined as a bioavailability between 0% and 30%, intermediate bioavailability was defined as a bioavailability between 30% and 70% and high bioavailability was defined as a bioavailability between 70% and 100% (Center for Drug Evaluation and Research, 2022; Kim et al., 2014). This classification facilitates risk assessment and guides subsequent steps in the evaluation process. Infant systemic exposure depends not only on the amount of the medication that reaches the infant’s systemic circulation but also on the infant’s ability to eliminate (i.e., metabolize and/or excrete) the medication and whether there is a risk of accumulation. Accumulation is defined as the relationship between the dosing interval and the rate of medication elimination. The accumulation ratio (AR), derived from the medication’s half-life and dosing interval, is a key indicator of medication accumulation (Meineke and Gleiter, 1998). It can also be calculated by comparing the Area Under the Curve (AUC) after multiple doses to the one after a single dose. An AR equal to 1 indicates no accumulation (Li et al., 2013). This ratio should be calculated using the medication’s half-life in adults and the dosing interval prescribed to the mother. If there is a risk of medication accumulation in the mother, infant systemic exposure may vary depending on whether the medication is utilized intermittently, such as in fertility treatments (i.e., few days every month), or for a short-term period, such as with antibiotics (i.e., days to a few weeks), or for a long-term period such as with antidepressants or immune-modulators (i.e., months to years).

Most of the time, information on oral medication bioavailability in neonates and infants is unavailable. To address this, one option is to estimate infant bioavailability based on adult data and the physiological characteristics of infants, if achievable. In this decision tree, for feasibility purposes, the exposure risk for breastfed infants was determined based on oral bioavailability in adults, assuming that infant bioavailability will be 100%, or at least as high as in adults. Due to the lack of specific information, we generally adopt a more cautious approach, increasing the risk assessment for infants. The potential for medication accumulation and treatment duration should also be considered, based on oral bioavailability in adults.

Since many medications are not administered orally, oral bioavailability might not always be available. In the absence of oral bioavailability data in adults, the medication’s physicochemical properties and *a priori* knowledge of its bioavailability should be used to assess infant systemic exposure. For example, monoclonal antibodies are known to have a low bioavailability, which should limit infant systemic exposure except perhaps in premature neonates during the early neonatal life (Ovacik and Lin, 2018). However, the risks associated with intestinal exposure should, in this case, also be evaluated.

Similarly to the medication safety profile, a conservative approach should be used when evaluating the expected level of medication exposure, always choosing the highest risk category in case of uncertainty.

### 2.2 Step by step application of the Milk4baby decision tree

The Milk4baby decision tree is a combination of the three factors detailed above, as presented in Figure 1. Algorithms were constructed for each factor and are further detailed in the following section. Each factor must first be categorized individually before being combined to select the most pragmatic and contextualized methodological approach for assessing infant safety profile following systemic exposure during lactation. An online interactive version of each algorithm is available at: https://www.entis-org.eu/milk4baby-decision-tree.

When evaluating each factor, if uncertainty arises between two groups, the most conservative category should be selected. When considering prevalence, researchers should assume a lower recruitment of participants rather than a higher one. Conversely, when evaluating medication safety profile or exposure level, the highest risk category should be considered.

#### 2.2.1 Evaluation 1: Expected prevalence of medication utilization in the childbearing population

**FIGURE 2:**
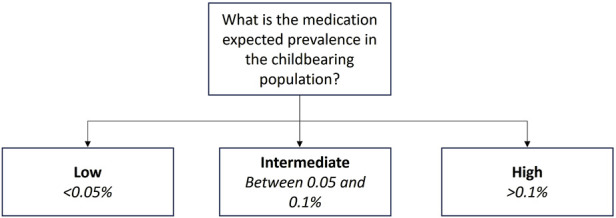
Evaluation of the expected prevalence of utilization in the childbearing population.

Information on the prevalence of medication utilization in the childbearing population, or in the general population can be retrieved from scientific articles or medication prescription databases such as the Agence Nationale de Sécurité du Médicament et des produits de santé (ANSM), the Medicines and Resources Information Project (GIP) databank or pregnancy databases such as EFEMERIS (Evaluation chez la femme enceinte des médicaments et de leurs risques) (data ansm, 2020; GIPdatabank, 2024; EFEMERIS, 2005). Some countries have their own medication prescription database; therefore, the expected prevalence of medication utilization in the childbearing population should be evaluated based on data obtained from the country where the lactation study will be conducted or from a country with similar prescription patterns. For example, medication prescription databases for European countries were documented by Ballarín et al. (2015). When a medication has multiple indications, the most common indication should be used to estimate its expected prevalence in the childbearing population. Based on the information collected from the literature or databases and the classification presented in Section 2.1.1, one can determine whether a medication has an expected low, intermediate, or high prevalence in the childbearing population. Figure 2 illustrates this initial step.

#### 2.2.2 Evaluation 2: Medication safety profile

To evaluate the safety profile of a medication, regardless of the population, it is necessary to review published studies on adverse effects, clinical studies, as well as pharmacovigilance, pharmacoepidemiology, and post-authorization studies (European Medicine Agency, 2024a; Sabaté and Montané, 2023; European Medicine Agency, 2024b). These studies should assess the type, severity, and frequency of adverse effects related to the medication. Additionally, information on the safety of the medication in young infants can be obtained from the product monograph, particularly the pediatric section, and national databases such as SwissPedDose and MICROMEDEX (SWISSPEDDOSE, 2024c; Micromedex, 2024). A recent review mapped all neonatal medication formularies available to provide information on neonatal medications (Shaniv et al., 2023). These resources help determine if the medication can safely be utilized in young infants.

To confirm that medication’s safety in infants has been established, clear information on the medication’s adverse effects in this population must be available. For instance, acetaminophen is well-studied and approved for neonates born from 28 weeks of gestational age with multiple studies published on its utilization in this age group (SWISSPEDDOSE, 2024b; Anderson et al., 2002). Conversely, if no studies on the medication’s adverse effects in infants exist, its safety has not been determined for this specific population. For example, citalopram is only approved for children from the age of seven, so it would not be considered safe for infants (SWISSPEDDOSE, 2024a). If a medication is safe in infants, as defined in Section 2.1.2, the safety risk should be low; if its moderately safe, then it should be intermediate and if it is unsafe, it will be high.

**FIGURE 3:**
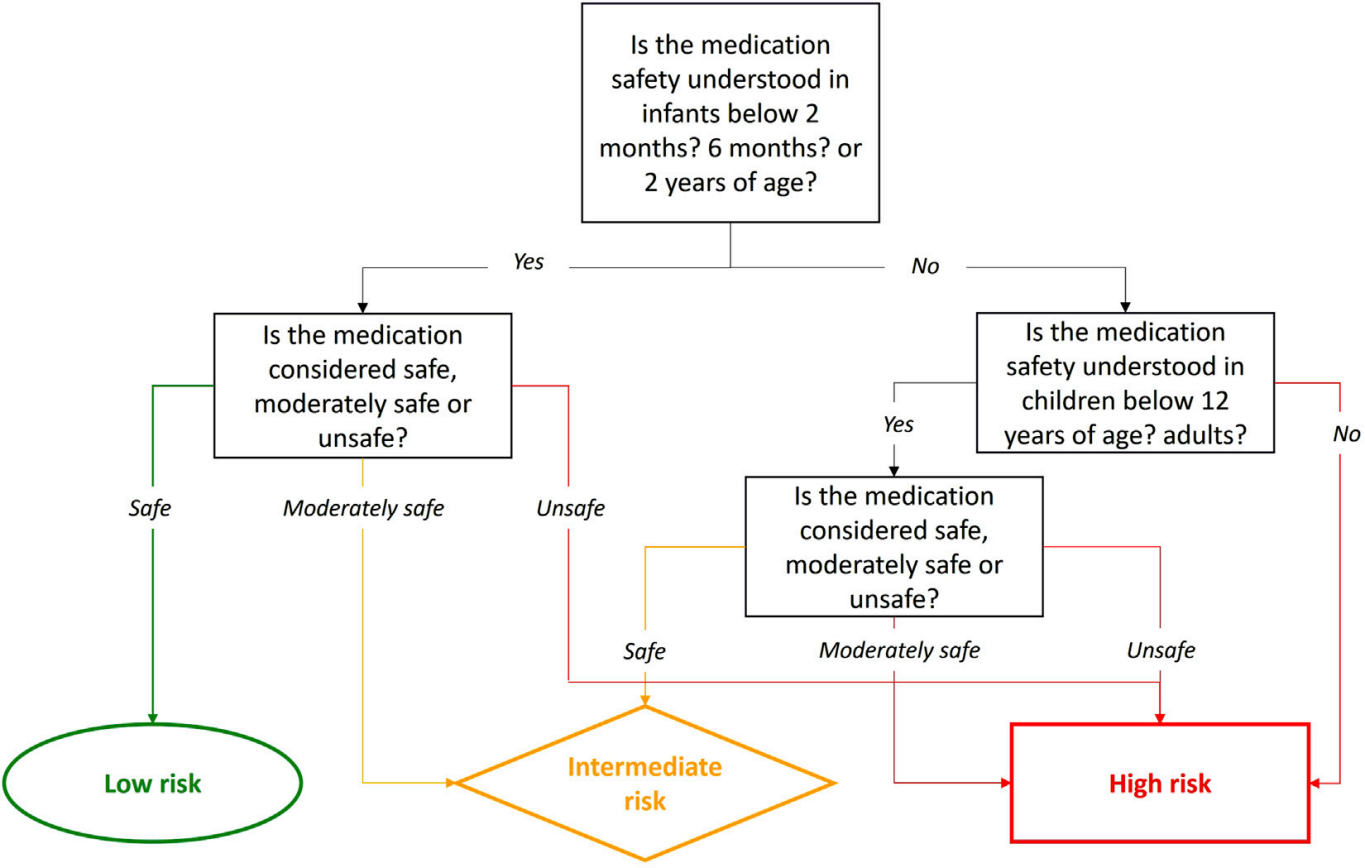
Evaluation of the medication safety profile in infants.

Similarly, if clinical, pharmacovigilance, or pharmacoepidemiology studies indicate the medication’s safety in adults, such as with citalopram, which has a well-known safety profile in adults, the medication’s safety should be considered established for adults (Muldoon, 1996). However, the lack of information on the medication’s safety in infants led us to increase the safety risk for a breastfed infant. Consequently, the risk for the infant would be intermediate if the medication is safe and high if the medication is moderately safe or unsafe for adults. Figure 3 depicts the step-by-step evaluation of the medication safety profile for breastfed infants.

A high-risk category indicates that a thorough evaluation of infant systemic exposure during lactation is warranted. Along with the selected methodological approach, the high-risk category will influence the study design, i.e., the type and number of samples collected, for example. Details on specific study design parameters to consider are presented later in this paper.

#### 2.2.3 Evaluation 3: Level of medication exposure in infants

Medication exposure level information can be obtained from clinical pharmacokinetic studies, monographs, and national databases such as Micromedex and Swissmedicinfo (Micromedex, 2024; Refdata, 2024).

The evaluation of the expected infant’s level of exposure to a medication during lactation begins with assessing its bioavailability in the mother. If the medication is not bioavailable or has a limited bioavailability (e.g., topical forms), the infant’s exposure risk is automatically considered low, as the medication cannot reach human milk. However, if the medication reaches therapeutic systemic levels in the mother, the potential for transfer into human milk in high amounts must be evaluated. Medications with high transfer potential may increase the infant’s exposure risk. The next step is to assess the oral bioavailability in the infant. Low oral bioavailability in infants generally results in a low exposure risk, whereas intermediate or high oral bioavailability may increase the exposure risk. A lack of oral bioavailability data, either in infants or in adults, also raises the risk level due to the uncertainty. Lastly, the frequency and duration of utilization play an important role. Intermittent or short-term utilization is typically associated with a lower exposure risk than long-term chronic utilization, especially for medications with a potential for accumulation. Even when a medication demonstrates limited transfer into human milk or a low oral bioavailability, the possibility of accumulation in the infant must be carefully considered, as it could still pose a danger over time.

**FIGURE 4:**
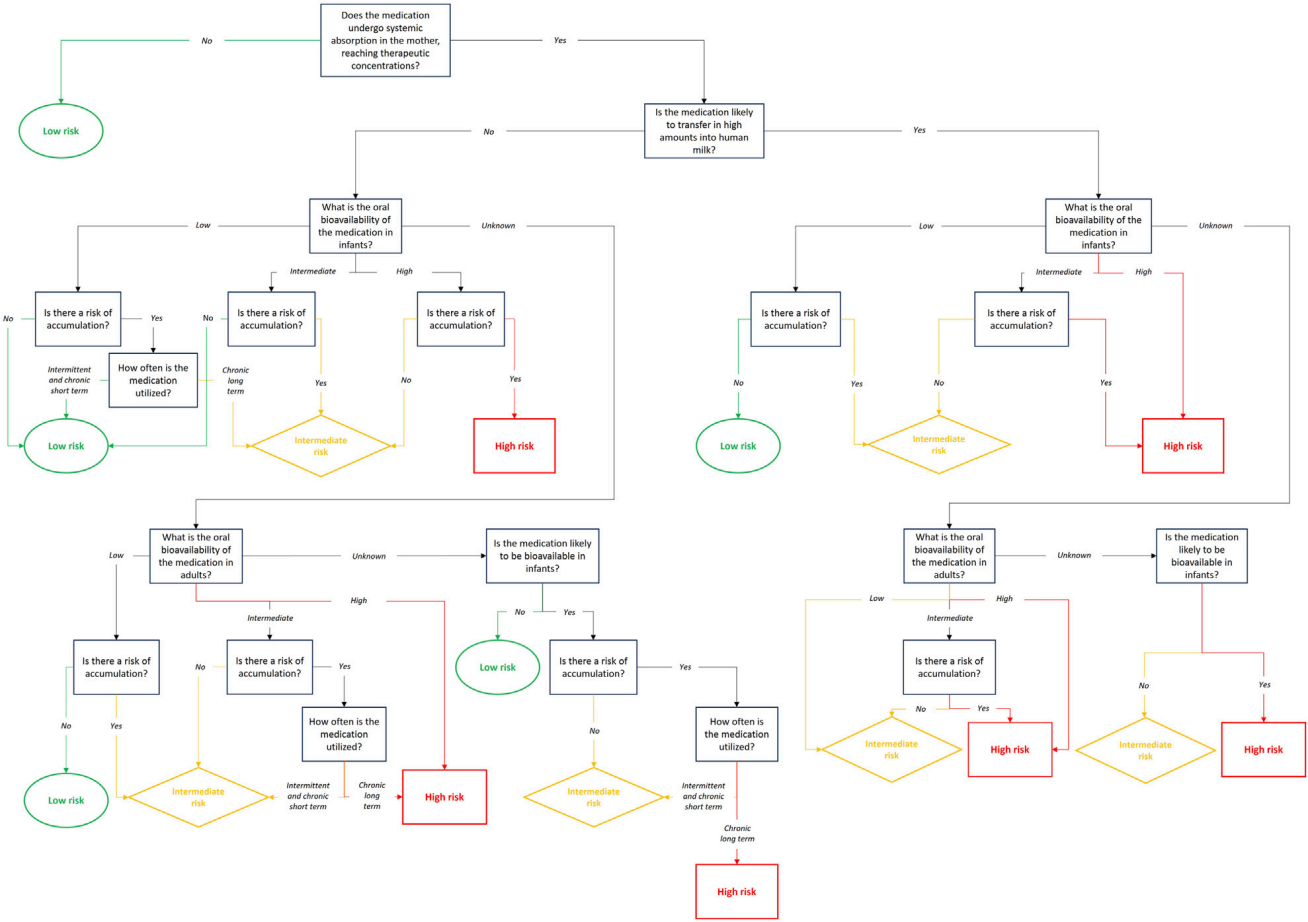
Evaluation of the infant medication level of exposure.

Figure 4 shows the step-by-step evaluation of the expected level of infant exposure, based on maternal and infant patterns.

#### 2.2.4 Methodological approach selection

Once the expected prevalence of medication utilization in the childbearing population, the safety profile, and the exposure level in infants were determined, we developed a decision tree to guide the selection of the most pragmatic and contextualized methodological approach between case reports, PBPK, popPK and pharmacoepidemiologic studies. In this context, case reports should specifically provide information on infant exposure, including at least medication concentrations in breast milk samples and clinical outcomes in infants. With such approach, the quality and impact of case reports can be improved. Further details on the recommended content of these case reports are provided in Section 4, in accordance with the recommendations of Anderson (2022). The prevalence of medication utilization will influence the number of women available for clinical analyses, and thus the methods to use. If the expected prevalence is low, only a few women will be available to provide biological samples. In such cases, a popPK approach would not be feasible, but case reports/case studies and a PBPK approach might be more suitable. For medications with expected intermediate prevalence, a reasonable number of women could be recruited and provide blood and/or human milk samples allowing for the consideration of a popPK approach. However, with a small sample size, the popPK approach might not adequately characterize inter-individual variability, decreasing our confidence in the estimates. Therefore, we strongly recommend combining the top-down and bottom-up approaches (i.e., popPK and PBPK models) to enhance confidence in the estimates, especially in high-risk cases. If the medication is highly prevalent, and many women can be recruited, the popPK approach, and potentially pharmacoepidemiologic studies for high-risk cases, would be the most pragmatic and contextualized methodologies. High-risk cases are determined using the safety profile and exposure level algorithms. Therefore, here are our recommendations for the methodological approach to use based on the 3 factors we evaluated, i.e., the expected prevalence of medication utilization in the childbearing population, the safety profile and the level of exposure.

**FIGURE 5:**
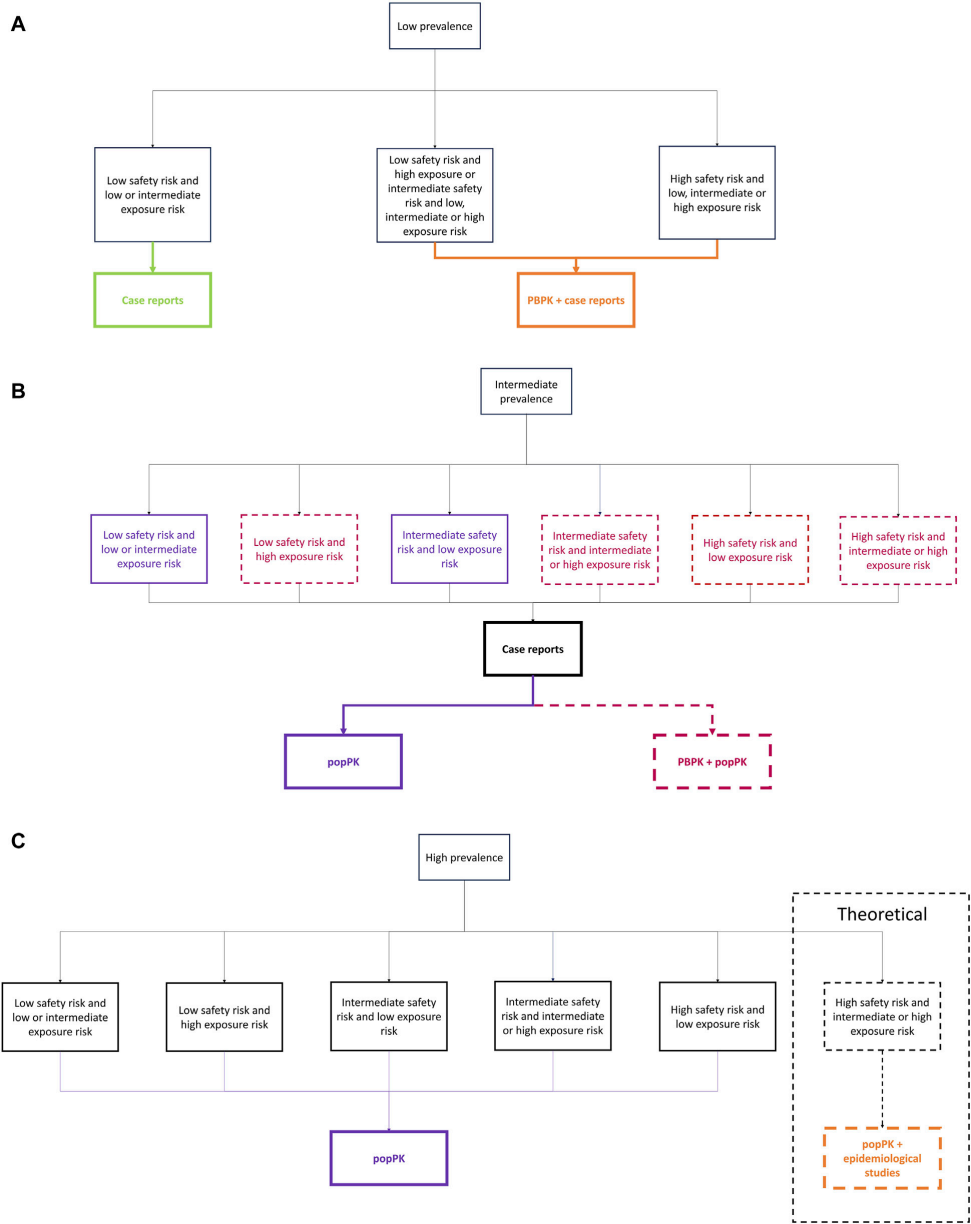
Decision tree for **(A)** low prevalence, **(B)** intermediate prevalence, and **(C)** high prevalence.

If the medication expected prevalence is low (Figure 5A), and there is a low safety risk along with low to intermediate exposure risk for the infant, case reports or case studies are sufficient to evaluate the infant’s safety profile after systemic exposure during lactation. In this scenario, pharmacokinetic models are not recommended or feasible. Indeed, they would be time and resource consuming without being able to identify a potential risk for the breastfed infant. However, if there is a low safety risk but a high exposure risk, or an intermediate or high safety risk with any exposure risk, PBPK models become necessary. These models can predict variables such as inter-individual variability, that case reports cannot, and can therefore identify potential high exposure or safety risks. In such cases, case reports or case studies should complement the PBPK approach, with clinical data used to qualify the PBPK predictions.

If the expected prevalence of the medication is intermediate (Figure 5B) and it has a low safety risk and a low or intermediate exposure risk, popPK models are adequate. In the absence of safety or exposure risks, popPK models with a small number of subjects are acceptable to estimate the general infant safety profile after systemic exposure without compromising infant safety. However, if the safety risk is low but the exposure risk is high, both popPK and PBPK models should be employed to evaluate infant systemic exposure, as higher confidence in the results is needed due to the potential risk to the infant. When the safety risk is intermediate and the exposure risk is low, popPK models are again most adequate. However, if the safety risk is intermediate and the exposure risk is either intermediate or high, both popPK and PBPK models should be used to account for the increased risk to the infant. In cases with a high safety risk, regardless of the exposure risk, both popPK and PBPK models are necessary to thoroughly evaluate the infant’s exposure during lactation and the associated risks. In all cases, case reports/case studies should serve as preliminary data due to the time required to complete pharmacokinetic studies. They should also be used to enhance confidence in the results obtained through modeling and simulation approaches. If sufficient experimental data is available, PBPK models can further inform study design, such as optimizing sampling strategy for subsequent popPK models.

If the medication expected prevalence is high (Figure 5C), popPK should always be used. Pharmacoepidemiologic studies should be considered in combination with popPK for medications with a high safety and exposure risk for infants. However, this scenario is largely theoretical, as it is unlikely that a medication with a high safety and exposure risk would be frequently prescribed to lactating women. It is highly probable that no medication will fit into this category. Although case reports/case studies are not the most suitable study type for prevalent medications, it is important to note that popPK studies can take time to recruit a sufficient number of women. Therefore, case reports/case studies should be used to present preliminary results on the infant safety profile following systemic exposure to the medication during lactation. Similarly, PBPK models can be used to inform the design of popPK studies or pharmacoepidemiologic studies.

## 3 Verification of the Milk4baby decision tree

### 3.1 Preliminary application

A preliminary application was conducted to illustrate how to use the Milk4baby decision tree. Citalopram, methotrexate, amiodarone, infliximab, cysteamine and acetaminophen were selected due to their varying toxicity, physicochemical properties, and pharmacokinetic profile. Supplementary Figure S1 shows how the algorithms were used to determine the prevalence of medication utilization, as well as the safety and the exposure risks, for citalopram.

Selective serotonin reuptake inhibitors (SSRIs) are frequently utilized during pregnancy and lactation to treat depression and other mental health conditions. The prevalence of SSRIs during the year following delivery is 4.66%. Specifically, citalopram is utilized by 0.77% of pregnant women (Molenaar et al., 2020). It is likely that the prevalence of citalopram utilization during lactation is similar to or higher than this rate. Therefore, citalopram can be classified in the high expected prevalence category. The safety of citalopram in children younger than 7 years old is unknown (SWISSPEDDOSE, 2024a). However, its safety profile in adults is well-established. Adverse effects in adults are generally considered mild to moderate and include nausea, dry mouth, somnolence, increased sweating, and diarrhea (Muldoon, 1996). Thus, the safety risk of citalopram is considered intermediate. The pharmacokinetics of citalopram have not been evaluated in children younger than 7 years old, so oral bioavailability in neonates and infants is also unknown. In adults, the medication’s oral bioavailability is 80%, indicating systemic absorption in the mother. Based on the physicochemical and pharmacokinetic characteristics of citalopram, it is likely to transfer into human milk in high amounts. Citalopram is typically administered once daily and has a half-life of 35 h (Sharbaf Shoar and Padhy, 2023). An accumulation ratio of 2.56 can be calculated for this medication, indicating a risk of accumulation in neonates and infants. Additionally, citalopram is usually administered chronically for long-term periods further increasing the risk of exposure for neonates and infants if the mother is taking this medication. Based on our decision tree, the best methodological approach would be popPK. Given the high risk of exposure for neonates and infants, blood samples from infants should be considered to evaluate the risk of accumulation due to their limited capacity to eliminate the medication.

As mentioned in the introduction, methotrexate is classified in the intermediate expected prevalence category. Indeed, it is frequently utilized in the general population but contraindicated in pregnant women due to its teratogenic effects (Dawson et al., 2014). In lactating women, methotrexate is often utilized off-label to treat ectopic pregnancy (Wang et al., 2024). Based on this information, methotrexate can be considered as an intermediate expected prevalence medication. Methotrexate’s safety has been evaluated in children under 2 years old. According to the literature, it demonstrates a good safety profile, with adverse effects such as anemia, gastrointestinal issues, joint pain, weakness, and liver enzyme elevation occurring in 57.1% of patients (Barak Levitt et al., 2023). All these adverse effects are dose-dependent, indicating an intermediate safety risk for methotrexate. Methotrexate is unlikely to transfer in high amounts into human milk due to its high molecular weight and hydrophilicity. In children aged 1.9–18 years, the oral bioavailability of methotrexate is 33%, categorizing it as intermediate (Teresi et al., 1987). The accumulation ratio is 1, as methotrexate is typically only administered once intramuscularly for ectopic pregnancy or weekly for other indications. Its half-life ranges from 5 to 8 h, resulting in an intermediate exposure risk (Bannwarth et al., 1996). Based on the decision tree, the most appropriate methodological approach would be popPK and PBPK. Case reports should be utilized as preliminary results to ensure the safety profile of methotrexate during lactation.

Amiodarone is a frequently prescribed medication, with prescription prevalence of over 0.1% in England and the United States, utilized to treat arrythmias (Hayward et al., 2015; Vassallo and Trohman, 2007). However, amiodarone is known to have adverse effects on the fetus during pregnancy and is currently not recommended for utilization during pregnancy and lactation (Chow et al., 1998). Therefore, the expected prevalence of amiodarone utilization in the childbearing population is considered intermediate. Amiodarone is prescribed to children younger than 28 days and has been shown to be safe in those under 2 years old. The adverse effects in this age group are generally mild and transient, not necessitating withdrawal of the medication (Perry et al., 1996; Etheridge et al., 2001). Consequently, the safety risk for amiodarone is considered low. Amiodarone is highly lipophilic and has a very long half-life, which makes it likely to transfer into human milk in high amounts. In children, amiodarone has an oral bioavailability of around 50% which is classified as intermediate. Its half-life in adult is 53 days, but it has been reported as 14 days in a 28-day-old child (Buck, 2001). Amiodarone is typically administered once daily, leading to an accumulation ratio of at least 20, indicating a very high risk of accumulation. Additionally, amiodarone is utilized chronically for long-term periods, resulting in a high exposure risk for infants. Therefore, the most appropriate methodological approaches for evaluating amiodarone safety in breastfed infants would be popPK, PBPK, and case reports/case series, with case reports/case series serving as preliminary results. Due to the high risk of amiodarone accumulation in infants, infant blood samples should be considered to assess the level of exposure.

Infliximab is a monoclonal antibody utilized to treat conditions such as Crohn’s disease and rheumatoid arthritis, typically as a secondary line treatment. Crohn’s disease has a prevalence of approximately 0.1%–0.2% in north America and Europe, respectively, and is the most common indication for infliximab (Loftus, 2004). Therefore, the expected prevalence of infliximab utilization can be considered intermediate. The safety of infliximab has been demonstrated only in children older than 3 years, where it is regarded as safe with mild and infrequent adverse effects. Typical adverse effects include shortness of breath, throat tightening, chest pain, and rash. It is also considered safe in adults (Friesen et al., 2004; Northcutt et al., 2014; O’Donnell et al., 2011). Consequently, infliximab is categorized with an intermediate safety risk. The bioavailability of infliximab in children is unknown, but it is administered intravenously in adults, where it achieves 100% bioavailability in the bloodstream. However, some literature suggests that antibodies, including infliximab, typically have low oral bioavailability (1%–2%) due to their size and their vulnerability to stomach acidity and intestinal enzymes (Ovacik and Lin, 2018). For the same reasons, infliximab is unlikely to transfer into human milk in significant amounts. The half-life of infliximab ranges from 11 to 19 days, and it is usually administered every 8 weeks in adults for Crohn’s disease (Hemperly and Vande Casteele, 2018). The calculated AR of 1.07 suggests minimal risk of accumulation. Based on this information, the exposure risk for infliximab is considered low. According to our decision tree, the most appropriate methodological approaches would be popPK and case reports as preliminary results.

Cysteamine is a medication utilized to treat nephropathic cystinosis, an extremely rare autosomal recessive metabolic disorder with a prevalence of 1.6 per million (0.00016%) (Langman et al., 2016). It can be classified in the low expected prevalence category. Cysteamine is approved for utilization in children older than 1 year. Very common adverse effects include vomiting, nausea, diarrhea, loss of appetite, fever, and drowsiness (European Medicines Agency, 2024e). The FDA has concluded that there is no evidence of safety concerns for cysteamine in pediatric populations (Food and Drug Administration, 2018). However, because the safety of the medication has not been evaluated in neonates and infants younger than 1 year, who are more at risk of exposure to cysteamine, the safety in this age group cannot be confirmed. Consequently, the safety risk is considered intermediate. Literature suggests that the oral bioavailability of cysteamine could be less than 10%, which corresponds to the low bioavailability category (Tennezé et al., 1999). Cysteamine is a small molecule with intermediate protein binding, suggesting that it could transfer in high amount in human milk. The half-life of cysteamine is 3.7 h following immediate release and 4.8 h after sustained release (Ariceta et al., 2024). It is typically administered two or four times daily, resulting in an AR between 1.1 and 1.7, suggesting a risk of accumulation. Therefore, cysteamine should be classified in the intermediate exposure risk category. Due to low expected prevalence of use of cysteamine and the intermediate safety risk, PBPK and case reports would be the most appropriate methodological approaches to evaluate infant safety profile following systemic exposure to this medication through lactation.

Acetaminophen is frequently utilized for pain relief and fever reduction and is the most commonly utilized over-the-counter medication among pregnant and lactating women, with more than 50% of women using it during pregnancy (Naseri et al., 2022). It is, therefore, highly prevalent in the childbearing population. The safety of acetaminophen has been evaluated in neonates from birth, demonstrating a good safety profile, which classified it as a low safety risk medication (Cuzzolin et al., 2013). Its bioavailability in young infants is around 72%, indicating high systemic exposure in this age group (Kleiber et al., 2019). The medication’s half-life ranges from 1.5 to 2.5 h (Mazaleuskaya et al., 2015). Typically administered every 4–6 h, the accumulation ratio for acetaminophen ranges between 1.06 and 1.49, suggesting a small risk of accumulation. Moreover, acetaminophen has several characteristics of a medication that can transfer into human milk in high amounts. Based on this information, acetaminophen may present a high risk of exposure. The most appropriate methodological approach for evaluating acetaminophen’s safety profile in breastfed infants would be popPK, according to our decision tree.

### 3.2 Extended verification of the Milk4Baby decision tree

The decision tree was verified by testing 50 medications randomly selected from the medications listed in LactMed and Le CRAT (Centre de Référence sur les Agents Tératogènes) databases. This process ensures that all types of medications are tested in the algorithm, preventing a selection bias. Non-pharmaceutical compounds such as botulin A or alcohol were intentionally excluded from the list as the decision tree was only developed for pharmacotherapeutic compounds. The verification process helped optimize the algorithm steps and demonstrate the tree’s effectiveness in identifying appropriate PK analysis methods. To ensure the robustness of the extended verification, JM and M-CH independently reviewed a random selection of medications from this list.

During the verification, the algorithms were iteratively refined. Initial testing revealed that some decision points in the algorithm left questions unanswered for specific drugs. We therefore adapted the algorithm step-by-step until all 50 medications could be processed. While formal quantitative criteria to definitely confirm the algorithm’s accuracy were not established, we considered the verification successful based on several factors: the ability to fully process all medications without unanswered questions, the consistency of recommendations between independent reviewers, and validation of the selected methods by project experts. The results of this verification are presented in Supplementary Table S1. In most cases (29 medications), due to the low expected prevalence of medication utilization in the childbearing population, and the presence of either a safety or exposure risk, the Milk4baby decision tree identified PBPK models and case reports as the most appropriate methodological approaches to assess infant safety profile following systemic exposure during lactation. PopPK was the second most frequently recommended method according to our decision tree.

## 4 Design of clinical lactation studies

Once all the key factors have been evaluated and the most pragmatic and contextualized methodological approach has been selected, additional components of the study design must be carefully considered to develop a robust lactation study including clinical samples. Specifically, the following parameters should be considered, though not exclusively: the number of women-infant dyads to recruit, the types of biological samples to collect, the number of samples required and the medication pharmacokinetic variability. PBPK models can also be a useful tool for determining these components before designing the lactation study.

### 4.1 Number of women-infant dyads

The number of women to recruit for popPK or pharmacoepidemiology studies depends on the *a priori* pharmacokinetic inter-individual variability reported in the literature. If there is minimal or no inter-individual variability, fewer participants may be needed to assess variability in infant safety profile and systemic exposure during lactation. Conversely, in case of significant inter-individual variability with main covariates identified, it is crucial to recruit a sufficient number of women to account for all the possible variations. For example, if one of the significant covariates is the CYP2C19 phenotype and considering that CYP2C19 poor metabolizers represent 2%–5% of Caucasians, 5% of African Americans and up to 25% of Asians, then approximately 100 women should be recruited to include CYP2C19 poor metabolizers in a study including Caucasians or African Americans (Ibeanu et al., 1998). However, recruiting 100 women may not always be feasible. In such cases, an alternative would be to combine popPK and PBPK approaches to simulate poor metabolizers based on a model with extensive metabolizers and an adequate knowledge of the blood-milk barrier processes.

### 4.2 Number of samples

In designing a lactation study, the number of samples is also a crucial consideration. Various sampling schemes are available, including rich sampling, sparse sampling, and opportunistic sampling.

#### 4.2.1 Rich sampling

Rich sampling involves collecting multiple samples from each individual over a period of time to thoroughly assess how the body processes the medication. This method is useful for all types of pharmacokinetic studies, as it allows for a detailed characterization of the medication’s pharmacokinetics over the dosing interval. For example, the M/P ratio can vary over time after dosing and accurate evaluation of medication transfer into human milk requires calculating the AUC of medication concentrations in both maternal blood and human milk (Anderson, 2018). However, rich sampling can be restrictive and intrusive for participants and is therefore more often used for case reports and case series. It is also more commonly applied to human milk due to its noninvasive collection, compared to maternal or infant blood samples. For human milk, the number of samples collected generally depends on the infant’s drinking behavior, unless specific milk sampling time points are defined. In such cases, the concentrations measured may be less representative of the infant’s actual exposure.

#### 4.2.2 Sparse sampling

Sparse sampling involves collecting one or a few samples per individual. It is less invasive and burdensome for participants. This type of data can be analyzed using a popPK approach, which enables the evaluation of the medication’s pharmacokinetic profile across the population provided that a sufficient number of individuals are included. Sparse sampling is often conducted at random intervals, but in some cases, predetermining sampling times may be beneficial to capture specific phases, such as absorption or excretion. For human milk samples, it is more accurate to align sampling with feeding times to better capture infant systemic exposure. Sparse sampling is typically preferred for maternal blood samples and should always be used for infant samples due to ethical considerations (Howie, 2011).

#### 4.2.3 Opportunistic sampling

Opportunistic sampling is a practical approach where samples are collected concurrently with routine or clinical procedures, thus avoiding the need for additional invasive procedures solely for research purposes. Since collecting infant blood for lactation studies can be challenging due to its invasive nature, opportunistic sampling in infants should be utilized whenever available, particularly when there is a high safety or exposure risk for the infants.

### 4.3 Type of samples

#### 4.3.1 Milk samples

Collecting only milk samples is the most favored approach in lactation studies and is recommended by the FDA if there is no reason to conduct another type of study (Food and Drug Administration, 2019). When there is no high safety or exposure risk, a milk-only study should be sufficient to evaluate infant systemic exposure during lactation. However, it is important to note that this type of study do not capture altered pharmacokinetics in mothers, limiting the extrapolation of data and generating fewer mechanistic insights. Moreover, in our clinical experience, most mothers have no objection to providing blood samples as well.

##### 4.3.1.1 Types of milk samples

Milk composition and volume vary significantly over time and theses variabilities should be considered in clinical studies. Mature milk should be preferred, as colostrum and transitional milk might not accurately reflect medication transfer, except in specific cases where the medication is administered during the immediate neonatal period. In such cases, colostrum would be more appropriate if there are concerns about exposure, although the exposure through colostrum is very low due to the small volume of milk produced at this time. Both foremilk and hindmilk do not need to be collected for every lactation study; however, the study protocol should specify which type of milk is being collected and acknowledge that medication concentrations will vary depending on the milk composition (Food and Drug Administration, 2019). The FDA recommends collecting the entire milk volume from both breasts over 24 h to accurately evaluate infant systemic exposure to the medication, with the remaining volume given to the infant after aliquots are taken. Although more accurate, this method may be inconvenient for both the mother and the infant due to its complexity and can put lactation at risk.

#### 4.3.2 Milk and maternal plasma samples

Studies that include milk and plasma samples should be employed when the pharmacokinetics of the medication in lactating women is not well understood. This approach provides additional information about the amount of medication transferred into human milk. Such sampling schemes are particularly relevant when there is concern about medication accumulation in human milk. Therefore, if there is a risk of medication accumulation, plasma samples should be collected alongside milk samples. Ideally, milk and plasma samples should be collected at the same time to estimate the M/P ratio of the medication.

#### 4.3.3 Mother-infant pair samples

Based on FDA guidelines, mother-infant pair plasma samples should be considered when there is existing information about the extent of medication transfer into human milk, including evidence of medication accumulation in human milk and potential absorption by the breastfed infant. These plasma samples would also be useful when there is no information on medication transfer into human milk but a suspicion of high exposure in the infant. According to our decision tree, this would apply to medications with known intermediate or high bioavailability and a risk of accumulation, or if the medication’s oral bioavailability is unknown in infants, but low, intermediate, or high in adults with a risk of accumulation. Collecting infant blood may be challenging due to ethical concerns. In recent years, new collection methods have been developed to reduce both the volume of blood collected and the pain experienced by the infant. Indeed, dried blood spot (DBS) and Volumetrically Accurate Microsampling (VAMS) are particularly effective, with the latter offering greater precision (Protti et al., 2025; Wickremsinhe et al., 2023; Moorthy et al., 2021). One of the main applications of these techniques is to collect blood in neonates but it is also used for pharmacokinetic studies and therapeutic medication monitoring. However, they require validated DBS/plasma conversion methods (Londhe and Rajadhyaksha, 2020). These techniques hold great promise for the future of clinical lactation studies.

### 4.4 Medication pharmacokinetic variability

Inter-individual variability can affect how a patient responds to a medication, influencing both its safety and efficacy. This variability primarily impacts the pharmacokinetics of the medication and consequently, medication exposure. In lactation studies, this is particularly important as pharmacokinetic parameters can vary in both the mother and the infant. Therefore, once the most pragmatic and contextualized methodological approach is selected, inter-individual variability in pharmacokinetic parameters should be carefully considered when designing the clinical study, especially in popPK and pharmacoepidemiologic studies. Indeed, a higher number of participants should be recruited to better capture this variability when the medication is known to exhibit high inter-individual variability. To assess pharmacokinetic variability, the coefficient of variation (CV) for key parameters such as the area under the curve (AUC), clearance, volume of distribution and bioavailability should be considered. According to the literature, a CV lower than 30% indicates low PK variability, between 30% and 50% indicates an intermediate variability and more than 50% indicates a high variability (Food and Drug Administration, 2022; Jusko, 2017; Ågesen et al., 2019; Al-Sallami et al., 2014). Additionally, information on the factors contributing to the overall medication inter-individual variability should be collected.

### 4.5 Variables to collect

When designing a clinical lactation study, it is crucial to collect all variables necessary to assess infant safety profile and systemic exposure to the medication, as well as any covariates that might influence this transfer. Understanding the medication’s pharmacokinetics and its inter-individual variability is essential for this purpose. Anderson et al. have previously outlined the variables required for a case report, which are also fundamental for any lactation study (Anderson, 2022). The following variables should always be collected in lactation studies: maternal variables such as the age, weight, race or ethnicity, disease state and duration, comorbidities, infant variables such as the sex, weight, gestational age at birth, age at study participation, lactation status, and duration of lactation. Medication-related variables should cover the medications received by the mother (including dose, posology, route of administration, and timing of medication initiation), concentrations in any chosen matrix, time of medication intake, time of sample collection, and time of last feed. Lifestyle factors such as the consumption of alcohol, tobacco, and social or recreational drugs, adverse effects related information, and sample collection methods (e.g., foremilk, hindmilk, or entire volume, electric pump or manual collection) should be considered. In addition, other covariates should be collected if they are known to impact the medication’s pharmacokinetics. Examples include the timing of maternal meals, CYP450 phenotypes, comedications, or liver and kidney functions. Researchers often identify missing important variables only after analyzing study data. Therefore, careful consideration and planning of all potentially relevant variables should be done before the study begins.

## 5 Discussion

Recent experiences have shown that data collected in clinical lactation studies often lack robustness and exhibit high variability (Cardoso et al., 2023a). Case reports and case series dominate the field of lactation studies due to their opportunistic and straightforward approach. However, they are limited in assessing inter-individual variability among lactating women and even more so in explaining this variability. Moreover, case reports commonly will not provide information on patients with extreme characteristics (e.g., weight, renal function or CYP450 polymorphisms), unless it is an explicit aspect of the case report (Cardoso et al., 2023a). Similar limitations apply to classical pharmacokinetic studies. There is a clear need to improve and standardize clinical lactation studies, beginning with a more rigorous choice of methodological approaches to assess infant safety profile following systemic exposure to medication during lactation and inclusion of new methodological approaches, such as popPK and PBPK models, that can help overcome these limitations. The Milk4Baby decision tree developed in this study aims to support these improvements for medications in the post-marketing phase.

The Milk4Baby decision tree is based on key medication-related factors that should inform the choice of methodological approach: the expected prevalence of medication utilization in the childbearing population, as well as the medication’s safety profile and associated exposure risks. Each of these factors can be evaluated based on specific parameters established in the literature. However, accurately determining the expected prevalence of medication utilization, the safety profile and the expected level of exposure is complex and may not be feasible due to limited available data. As a result, several assumptions were made to estimate these key factors for the subsequent steps. These assumptions include, for example, that the safety risk of a medication is at least as high in infants as in adults, that medication bioavailability in infants is 100% or at least as high as in adults and that medication accumulation in infant is at least as high as in adults, when data for infants is lacking. However, these assumptions may not hold true in all cases. Similarly, if there is uncertainty between two categories, we consider that the higher risk category should be selected. These assumptions are intended to be conservative, representing a worst-case scenario to minimize any risk to breastfed infants. Although this decision tree presents a method for evaluating safety profile and exposure levels for breastfed infants, it is crucial to weigh these risks against the benefits of lactation. Indeed, lactation provides multiple benefits for infants, such as reducing the risk of various illnesses and death (Centers for Disease Control and Prevention, 2011b). Therefore, not lactating also increases the safety risk for infants.

By evaluating these three factors, one can determine the most pragmatic and contextualized methodological approach, between case reports/case studies, popPK, PBPK and pharmacoepidemiology, to assess infant safety profile following systemic exposure during lactation. Case reports/case studies should be preferred when the medication is not widely utilized and poses limited safety or exposure risk. Otherwise, case reports/case studies should serve as preliminary studies to provide initial data on infant systemic exposure or to verify PBPK models with clinical data when recruiting a sufficient number of women for popPK studies is not feasible. PBPK models are particularly recommended when medication utilization is rare or intermediate and there is a high potential for safety concerns or high exposure. Indeed, this modeling approach enhances exploring different scenarios and the impact on the pharmacokinetic concentration-time profile. However, PBPK models can be applied in almost all situations prior to lactation studies, as they also provide valuable insights for study design. PopPK studies should be conducted when medication expected prevalence is intermediate or high to ensure adequate sample size for evaluating the population variability in the pharmacokinetics. Pharmacoepidemiologic studies are only feasible when a large number of lactating women are taking the medication and are willing to participate in a clinical study. Due to the time and resources required, these studies should be reserved for situations with a high safety risk. However, given the risk for infants, it is rare for a highly toxic medication to be commonly utilized among lactating women. This scenario may seldom occur. Since pharmacoepidemiologic studies provide more information on adverse effects, they could later complement pharmacokinetic studies that focus more on exposure rather than on the actual effects of the medication.

By constructing this decision tree, we identified significant gaps in data related to infant safety profile and systemic exposure to medications. For example, pharmacoepidemiologic data on medication utilization during lactation are scarce, making it nearly impossible to accurately estimate the prevalence of specific medication utilization during lactation. The only way to approximate this prevalence is by examining databases on medication utilization for the general population and focusing on women of childbearing age. Recently, pregnancy registries, such as EFEMERIS in France and the Quebec Pregnancy Cohort (QPC) in Canada, have been implemented and provide valuable information (Benevent et al., 2019). These databases could be improved by collecting data on lactation stages or new databases could be created to specifically address the lactation population. Additionally, sparse measurements of medication concentrations in maternal plasma, human milk and infant blood could be collected and added to these databases to increase their pertinence. Many of these databases are not open access which may complicate access for evaluating the prevalence of medication utilization. We also observed that the pharmacokinetics and safety of many medications have not been evaluated in neonates and infants despite their utilization in these populations. Dosing is typically adjusted based on the infant’s weight or body surface area. However, pharmacokinetics in infants cannot be assumed to be the same as in adults, although it can be extrapolated based on factors such as age, height, weight, organ maturation and other physiological and ontogeny characteristics, as described by the ICH guidelines on pediatric extrapolation (European Medicines Agency, 2024c). For this purpose, PBPK modeling is a valuable tool, as it can distinctly describe physiological, and pharmacokinetic processes and predict infant systemic exposure during lactation without requiring a large dataset of clinical data for model development. Indeed, PBPK models can integrate multiple parameters, including medication-specific physicochemical properties, system knowledge related to pharmacokinetics and the physiology of pregnancy and lactation, as well as *in vitro* and *in vivo* data from both non-clinical and clinical experiments and clinical trial design (Manolis et al., 2024). Similarly, the decision tree suggested that PBPK should be used for 34 of the 50 medications tested during the verification process, highlighting its importance and potential impact in evaluating infant safety during lactation.

An important conclusion from our study is that, regardless of the recommended methodological approach, a minimum of clinical data will be necessary, either for model development or verification. Currently, this applies for PBPK models to evaluate their performance. However, as these models become more mechanistically refined, they hold the potential to allow predictions without the need for clinical data in the future. At present, women need to be recruited to provide blood, human milk or infant blood samples. Efforts should, therefore, focus on promoting recruitment and improving recruitment processes. As pharmacometric approaches are increasingly popular in studies involving pregnant and lactating women, designs using sparse sampling may be more feasible and accessible for women. However, rich sampling is often preferred to evaluate an individual’s pharmacokinetics more accurately, especially when recruiting a small number of women. For instance, it can be useful to calculate the M/P ratio based on AUC, rather than on a single time-point, to provide a more precise value. Nevertheless, this approach remains burdensome for the patient and does not capture inter-individual variability and should be limited to individual-level studies. Successful recruitment also relies on leveraging large collaborative research networks, which can help address low consent rate and provide essential research infrastructure. Real world data and big data in clinical research, retrieved from electronic medical systems, collect information on daily routine clinical practice and can inform on current clinical practice (Illamola et al., 2018). In recent years, applications and websites such as Meds4Mums2B in England, BELpREG in Belgium or Datamama in Switzerland, have been developed to gather data on pregnant women and improve access to medication safety information for this population (Alexe et al., 2024; Datamama, 2024; Sillis et al., 2023; Gerbier et al., 2023). Some of these applications also inform women about clinical research opportunities. In the near future, these tools should be expanded to include data collection and notifications for lactating women, raising awareness about research studies on infant systemic exposure during lactation. A recent study outlined strategies to recruit pregnant and lactating women for pharmacokinetic studies (Nakijoba et al., 2024). Key strategies include conducting community advisory board meetings to incorporate participant feedbacks into study design, protocols and informed consent forms, including peer mothers as co-investigators to address specific concerns and cultural contexts relevant to pregnant and lactating women, engaging with communities to build credibility and trust that can help to alleviate fears and misconceptions, establishing recruitment sites to facilitate participant enrolment, implementing safety protocols, and training research teams to communicate effectively and provide reassurance to participants. Stakeholder meetings, diverse communication platforms, and social media can further enhance recruitment efforts by broadening outreach to diverse groups (Rowland Yeo et al., 2025).

Efforts were made in the past years to change the perspective on including pregnant and lactating women in clinical trials, emphasizing the importance of collecting data on this special population. As one example, the EMA has recently acknowledged the need to enhance pregnancy and lactation labelling for medicines, as well as the potential of model-informed medication development (MIDD) approaches, especially PBPK, to improve labelling for special populations and increase confidence in enrolling these special populations in clinical trials (Manolis et al., 2024). The Medicines and Healthcare products Regulatory Agency also suggested the use of PBPK modeling to evaluate medication pharmacokinetics during pregnancy and lactation (Coppola et al., 2021). Other studies have also highlighted the usefulness of pharmacometrics approaches in addressing strict designs constraints associated with these special populations, such as limited subject numbers and restricted sampling opportunities. These approaches may also be the only viable methods for characterizing these populations, given their physiological differences and changes in metabolizing enzymes activity (Illamola et al., 2018). PopPK and PBPK have demonstrated their value in this context by estimating infant medication exposure via human milk while minimizing the number of samples collected from women. Both methodological approaches have shown their predictive performance to estimate infant systemic exposure, as demonstrated for medications like escitalopram or dolutegravir (Cardoso et al., 2023a; Ning et al., 2024; Dickinson et al., 2021). However, despite their many advantages, their use in lactation studies remains limited. The primary reason for this limited use is the poor understanding and quantification of system physiological parameters and mechanisms involved in mammary gland transfer (e.g., transporters at the mammary gland, surface area of the blood/milk barrier, unbound fraction in human milk), along with the physiological changes during lactation and in the maturing child (Van Neste et al., 2023). Additionally, there is a lack of characterization of the *in vitro* methods (e.g., permeability across the blood-milk barrier), necessary for quantitative prediction of medication transport mechanisms (Manolis et al., 2024). Promoting PBPK utilization would help establish proof of concept for these methods in lactation research. The more they are used, the more their performance will improve, and the less clinical data will be needed to develop PBPK with a good predictive performance. Moreover, further research should be conducted to enhance our understanding of the lactation process and the pharmacokinetics of medications in infants.

The high variability in published results is largely due to the lack of regulations governing clinical lactation studies. Additionally, the discrepancy between published literature, which often states that many medications are safe during lactation, and medication labels that caution against utilization during lactation, creates confusion for both lactating women and healthcare professionals. This discrepancy likely stems from regulatory agencies’ lack of confidence in the available real-life clinical data on medication safety during lactation to justify changing the labels and the absence of a requirement to update medication labels when new evidence becomes available. This highlights the need for regulatory agencies to establish guidelines for conducting clinical lactation studies that can reliably inform medication labelling. In this context, the EMA recently suggested revising its guideline on the Risk Assessment of Medicinal Products on Human Reproduction and Lactation: from Data to Labeling, citing a lack of clinical data on medication safety during lactation and the emergence of new modeling approaches (European medicines agency, 2024d). Additionally, lactation studies should be incorporated into regulatory requirements to accelerate the evaluation of medication safety during lactation. Finally, women sometimes receive conflicting advice from different healthcare professionals for the same medication, leading to confusion for lactating mothers. There is also a need to better educate healthcare professionals about lactation and the utilization of medication during this period.

With this decision tree, we aim to take a step forward in this direction by ensuring that the appropriate lactation study is developed for each specific medication. When used in the wrong context, case reports/case studies, popPK, PBPK and pharmacoepidemiologic studies can be inconclusive, leading to wasted time and resources. By using the Milk4baby decision tree, researchers can improve the efficiency and accuracy of determining infant safety profile following systemic exposure during lactation. Ultimately, this will allow to support better-informed decisions for both lactating women and their healthcare providers.

## Data Availability

The original contributions presented in the study are included in the article/Supplementary Material, further inquiries can be directed to the corresponding author.
