# Supplementary material for: Pragmatic and contextualized methods selection for safety assessment of infant systemic exposure through human milk: the Milk4baby decision tree approach - a contribution from the concePTION project
Source: Front Pharmacol. 2025 Aug 5;16:1602018. doi: 10.3389/fphar.2025.1602018 (PMC12361215; doi:10.3389/fphar.2025.1602018)
Supplement: Supplementary file 1 [file DataSheet1.docx]

Supplementary Material

**Pragmatic and Contextualized Methods Selection for Safety Assessment of Infant Systemic Exposure Through Human Milk: The Milk4baby Decision Tree Approach**

*A contribution from the concePTION Project*

**Anaëlle Monfort^1,*^, Julia Macente^2^, Martje Van Neste^3^, Miao-Chan Huang^2^, Nina Nauwelaerts^2^, Getahun Befirdu Abza^2^, Ursula Winterfeld^4^, Anne Smits^5,6,7^, Karel Allegaert^3,5,7,8^, Pieter Annaert^2,9^, Monia Guidi^10,11,12^, Alice Panchaud^1,13^**

*** Correspondence:** Anaëlle Monfort, anaelle.monfort@chuv.ch

**Supplementary Table 1.** Extended verification of the Milk4baby decision tree on 50 medications

| **Medication** | **Step 1** | **Step 2** | **Step 3** | **Model selection** |
| --- | --- | --- | --- | --- |
| **Adapalene** | High prevalence (1-3) | Intermediate safety risk (4-6) | Low exposure risk (5, 7) | popPK |
| **Amlodipine** | High prevalence (1-3) | Intermediate safety risk (8-10) | High exposure risk (11, 12) | popPK |
| **Azilsartan** | Low prevalence (1-3) | Intermediate safety risk (13-15) | Intermediate exposure risk (15) | PBPK + case reports |
| **Bendroflumethiazide** | Low prevalence (1-3) | Intermediate safety risk (16) | High exposure risk (17, 18) | PBPK + case reports |
| **Betamethasone**  **(topical)** | High prevalence (1-3) | Intermediate safety risk (19, 20) | Low exposure risk (21, 22) | PopPK |
| **Bisoprolol** | Intermediate prevalence (1-3) | Intermediate safety risk (23) | High exposure risk (24) | Case reports + PBPK + popPK |
| **Carfilzomib** | Low prevalence (1-3) | High safety risk (25, 26) | Low exposure risk (27) | PBPK + case reports |
| **Cefepime** | Low prevalence (1-3) | Low safety risk (28, 29) | Low exposure risk (29) | Case reports |
| **Ceftriaxone** | Intermediate prevalence (1-3) | Low safety risk (30, 31) | Low exposure risk (32) | Case reports + popPK |
| **Cidofovir** | Low prevalence (1-3) | Intermediate/high safety risk (33, 34) | Low exposure risk (35) | PBPK + case reports |
| **Clonidine** | Intermediate prevalence (1-3) | Low safety risk (36, 37) | High exposure risk (38, 39) | Case reports + PBPK + popPK |
| **Cyclopentolate (eye drops)** | Low prevalence (1-3) | Low safety risk (40-42) | Intermediate exposure risk (43-45) | Case reports |
| **Desonide**  **(topical cream)** | Intermediate prevalence (1-3) | Low safety risk (46) | Low exposure risk (47) | Case reports + PopPK |
| **Diflunisal** | Low prevalence (1-3) | Intermediate/High safety risk (48-50) | High exposure risk (51) | PBPK + case reports |
| **Dopamine** | Low prevalence (1-3) | Intermediate safety risk (52-54) | Low exposure risk (55) | PBPK + case reports |
| **Doxazosin** | Low prevalence (1-3) | Intermediate safety risk (41, 56) | Intermediate exposure risk (57) | PBPK + case reports |
| **Elagolix** | Low prevalence (1-3) | Intermediate safety risk (58) | Intermediate exposure risk (59, 60) | PBPK + case reports |
| **Entacapone** | Low prevalence (1-3) | Intermediate safety risk (61, 62) | Intermediate exposure risk (61) | PBPK + case reports |
| **Epoprostenol** | Low prevalence (1-3) | Low/intermediate safety risk (63, 64) | Low exposure risk (65) | PBPK + case reports |
| **Erdafitinib** | Low prevalence (1-3) | High safety risk (41, 66) | High exposure risk (67) | PBPK + case reports |
| **Escitalopram** | High prevalence (1-3) | Intermediate safety risk (41, 68, 69) | High exposure risk (70, 71) | popPK |
| **Filgotinib** | Low prevalence (1-3) | Intermediate safety risk (72-74) | High exposure risk (72, 75) | PBPK + case reports |
| **Furosemide** | Intermediate prevalence (1-3) | Intermediate safety risk (76-78) | High exposure risk (78) | Case reports + PBPK + popPK |
| **Gadoxetate** | Low prevalence (1-3) | Low safety risk (79) | Low exposure risk (80) | Case reports |
| **Interferon Gamma-1b** | Low prevalence (1-3) | High safety risk (81-83) | Low exposure risk (84) | PBPK + case reports |
| **Iothalamate** | Low prevalence (1-3) | Intermediate safety risk (85) | Low exposure risk (86) | PBPK + case reports |
| **Leucovorin** | Low prevalence (1-3) | Intermediate safety risk (87, 88) | High exposure risk (89, 90) | PBPK + case reports |
| **Levodopa** | Low prevalence (1-3) | Intermediate safety risk (41, 91, 92) | High exposure risk (93, 94) | PBPK + case reports |
| **Lidocaine** | High prevalence (1-3) | Intermediate/High safety risk (95, 96) | Low exposure risk (97, 98) | PopPK |
| **Lofexidine** | Low prevalence (1-3) | Intermediate/high safety risk (41, 99) | High exposure risk(100) (101) | PBPK + case reports |
| **Lonapegsomatropin** | Low prevalence (1-3) | Intermediate safety risk (102, 103) | Low exposure risk (103) | PBPK + case reports |
| **Mesalamine** | High prevalence (1-3) | Intermediate safety risk (41, 104) | Low/intermediate exposure risk (105, 106) | popPK |
| **Metronidazole** | High prevalence (1-3) | Low safety risk (107, 108) | High exposure risk (109) | popPK |
| **Migalastat** | Low prevalence (1-3) | Intermediate safety risk (110, 111) | High exposure risk (112) | PBPK + case reports |
| **Ocrelizumab** | Low prevalence (1-3) | Intermediate safety risk (113, 114) | Low exposure risk (114, 115) | PBPK + case reports |
| **Omeprazole** | High prevalence (1-3) | Low safety risk (116, 117) | Intermediate exposure risk (118, 119) | popPK |
| **Orphenadrine** | Low prevalence (1-3) | Intermediate safety risk (41, 120, 121) | High exposure risk (41, 48) | PBPK + case reports |
| **Phenazopyridine** | Intermediate prevalence (1-3) | Intermediate safety risk (122, 123) | High exposure risk (124) | Case reports + PBPK + popPK |
| **Phentermine** | Low prevalence (1-3) | Intermediate safety risk (125, 126) | High exposure risk (127) | PBPK + case reports |
| **Piperacillin and Tazobactam** | Low prevalence (1-3) | Low safety risk (128) (129, 130) | Low exposure risk (129) | Case reports |
| **Prochlorperazine** | Intermediate prevalence (1-3) | Intermediate safety risk (41, 131) | Intermediate exposure risk (132) | Case reports + popPK + PBPK |
| **Protriptyline** | Low prevalence (1-3) | High safety risk (133, 134) | High exposure risk (135) | PBPK + case reports |
| **Ribociclib** | Low prevalence (1-3) | Intermediate/high safety risk (136, 137) | High exposure risk (138) | PBPK + case reports |
| **Rituximab** | Low prevalence (1-3) | Intermediate safety risk (139-141) | Low exposure risk (115) | PBPK + case reports |
| **Siltuximab** | Low prevalence (1-3) | Intermediate/high safety risk (142-144) | Low exposure risk (115) | PBPK + case reports |
| **Ticarcillin and Clavulanic acid** | Low prevalence (1-3) | Low/intermediate safety risk (145-147) | Intermediate exposure risk (148) | PBPK + case reports |
| **Tobramycin** | Intermediate prevalence (1-3) | Low safety risk (94, 149) | Low exposure risk (150, 151) | Case reports + popPK |
| **Topotecan** | Low prevalence (1-3) | High safety risk (152-154) | Intermediate exposure risk (77, 155) | PBPK + case reports |
| **Tretinoin (Topical)** | High prevalence (1-3) | Intermediate safety risk (156-158) | Low exposure risk (159, 160) | popPK |
| **Umeclidinium** | Low prevalence (1-3) | Intermediate safety risk (161-163) | Low exposure risk (161, 164) | PBPK + case reports |

**Supplementary Figure 1**. Example of the algorithms use with Citalopram

**Evaluation 1:** Evaluation of the expected prevalence of utilization of Citalopram in the childbearing population


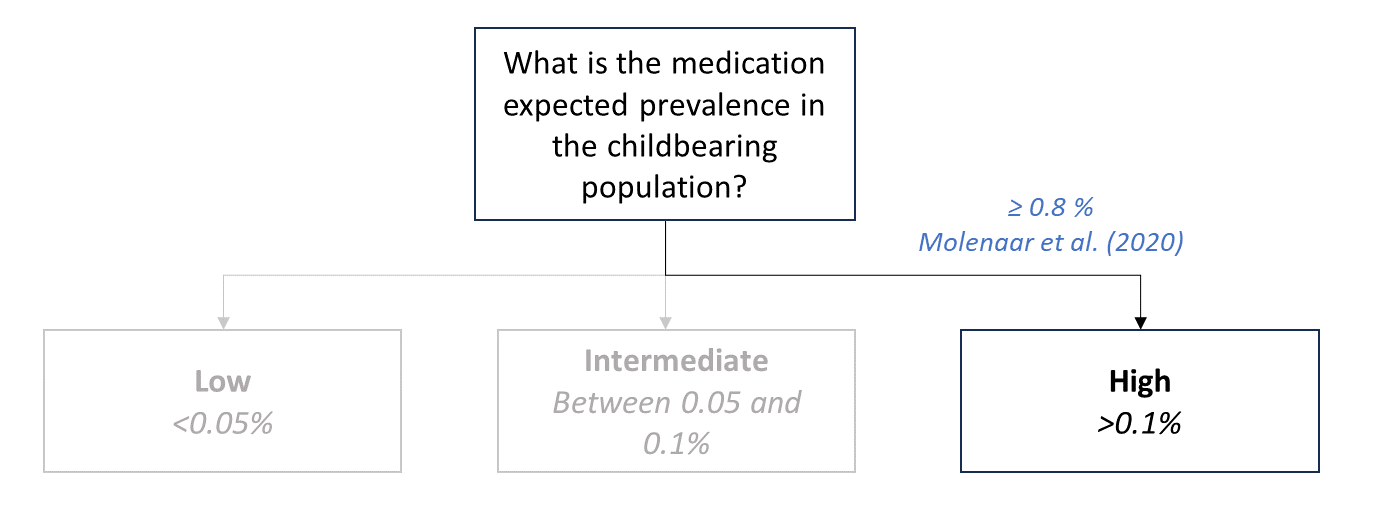


**Evaluation 2:** Evaluation of Citalopram safety profile in infants

**
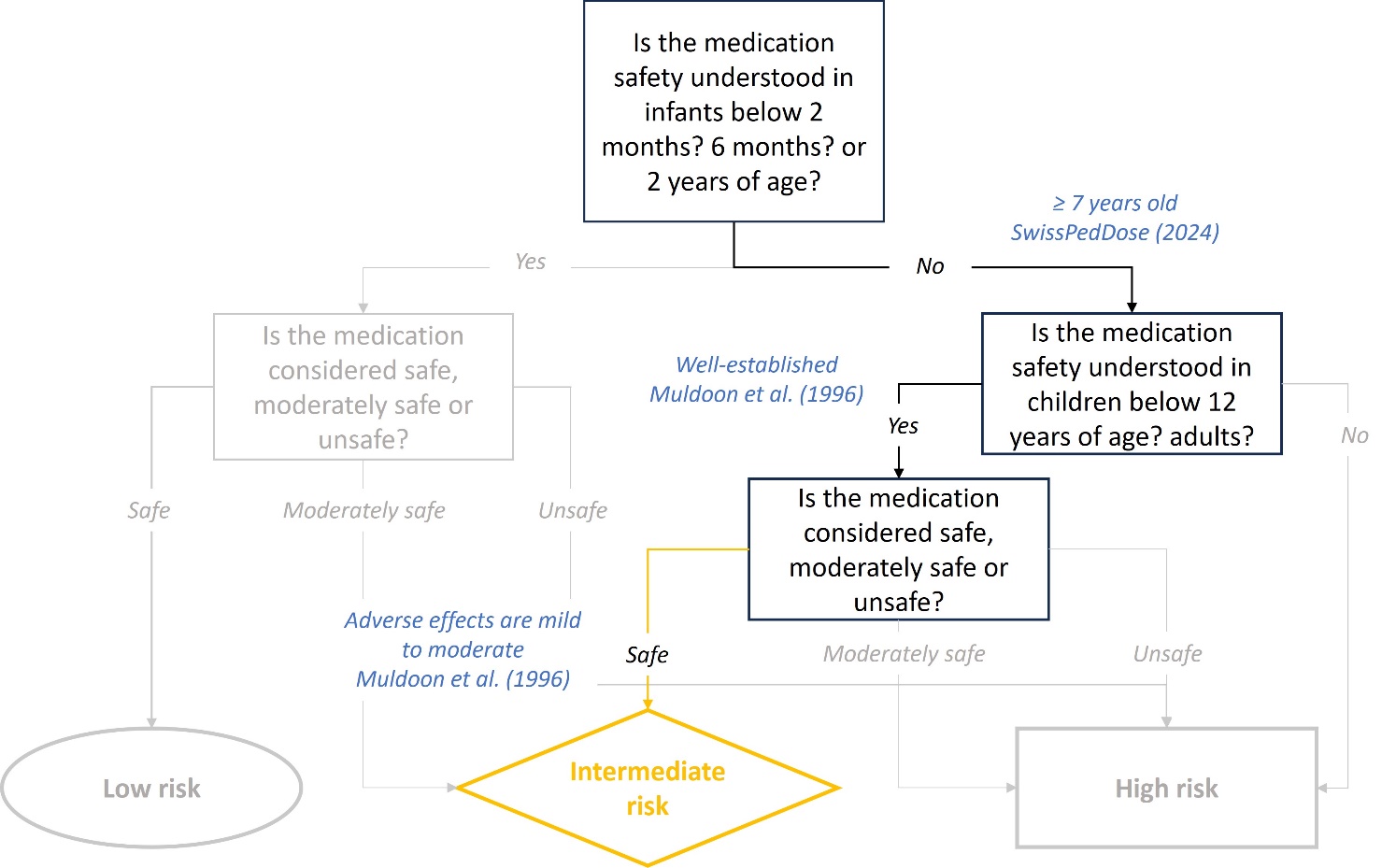
**

**Evaluation 3:** Evaluation of the infant level of exposure to Citalopram


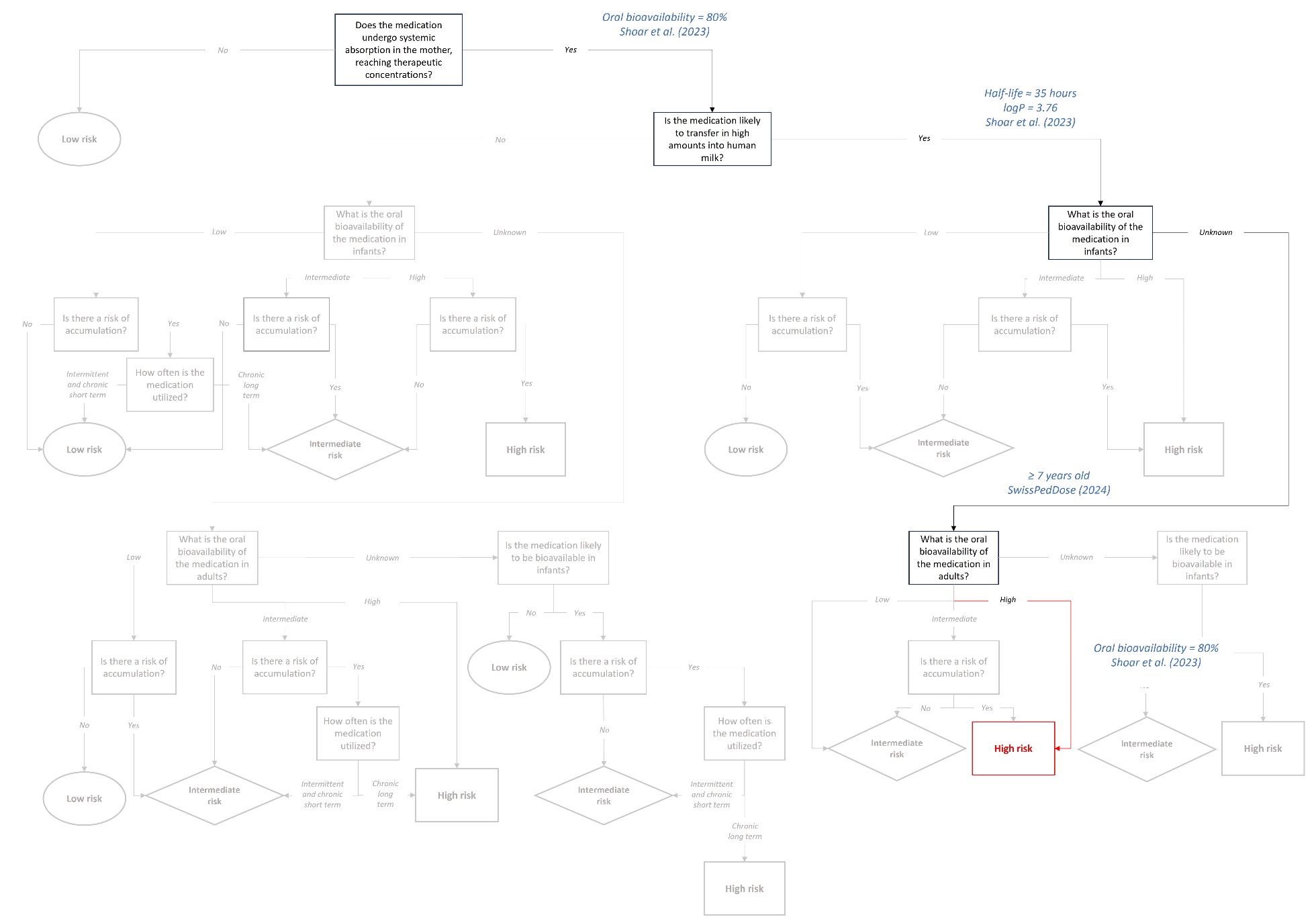


**References**

1. data.ansm [Internet]. 2020. Available from: <https://data.ansm.sante.fr/>.

2. The Norwegian Prescription Database (NorPD) [Internet]. 2021. Available from: <https://www.norpd.no/>.

3. GIPdatabank.nl [Internet]. Available from: <https://www.gipdatabank.nl/>.

4. Kose O, Koç E, Arca E. Adapalene Gel 0.1% in the Treatment of Infantile Acne: An Open Clinical Study. Pediatric Dermatology. 2008;25(3):383-6.

5. INC GC. PRODUCT MONOGRAPH - DIFFERIN. 2018.

6. Kose O, Koç E, Arca E. Adapalene gel 0.1% in the treatment of infantile acne: an open clinical study. Pediatr Dermatol. 2008;25(3):383-6.

7. Food and Drug Administration (FDA). PHARMACOLOGY REVIEW(S) - DIFFERIN. 2009.

8. Flynn JT. Efficacy and safety of prolonged amlodipine treatment in hypertensive children. Pediatr Nephrol. 2005;20(5):631-5.

9. Flynn JT, Newburger JW, Daniels SR, Sanders SP, Portman RJ, Hogg RJ, et al. A randomized, placebo-controlled trial of amlodipine in children with hypertension. J Pediatr. 2004;145(3):353-9.

10. Wang T, Wang Y, Lin S, Fang L, Lou S, Zhao D, et al. Evaluation of pharmacokinetics and safety with bioequivalence of Amlodipine in healthy Chinese volunteers: Bioequivalence Study Findings. Journal of Clinical Laboratory Analysis. 2020;34(6):e23228.

11. Bulsara KG PP, Cassagnol M. Amlodipine. StatPearls: Treasure Island (FL): StatPearls Publishing; 2024 [Available from: <https://www.ncbi.nlm.nih.gov/books/NBK519508/>.

12. Meredith PA, Elliott HL. Clinical pharmacokinetics of amlodipine. Clin Pharmacokinet. 1992;22(1):22-31.

13. Ito S, Nishiyama Y, Sugiura K, Enya K. Safety and efficacy of azilsartan in paediatric patients with hypertension: a phase 3, single-arm, open-label, prospective study. Clin Exp Nephrol. 2022;26(4):350-8.

14. European medicines agency (EMA). Assessment report - Edarbi. 2022.

15. European medicines agency (EMA). SUMMARY OF PRODUCT CHARACTERISTICS - Edarbi. 2015.

16. Rasmussen S, Borrild N, Vang Andersen J. Efficacy and Safety of 24 Weeks of Therapy with Bendroflumethiazide 1.25 mg/day or 2.5 mg/day and Potassium Chloride Compared with Enalapril 10 mg/day and Amlodipine 5 mg/day in Patients with Mild to Moderate Primary Hypertension. Clinical Drug Investigation. 2006;26(2):91-101.

17. Peri R, Mangipudy RS. Thiazide Diuretics. In: Wexler P, editor. Encyclopedia of Toxicology (Third Edition). Oxford: Academic Press; 2014. p. 539-45.

18. Authority HPR. Public Assessment Report for a Medicinal Product for Human Use - Bendroflumethiazide. 2020.

19. DeCastro M, El-Khoury N, Parton L, Ballabh P, LaGamma EF. Postnatal betamethasone vs dexamethasone in premature infants with bronchopulmonary dysplasia: a pilot study. Journal of Perinatology. 2009;29(4):297-304.

20. Schering-plough research institute. Celestone syrop 2006.

21. Täuber VU, Amin M, Fuchs P, Speck U. [Comparative studies in man on the percutaneous absorption of diflucortolone valerate, betamethasone-17-valerate, beclomethasone dipropionate and fluocinolone acetonide]. Arzneimittelforschung. 1976;26(7b):1492-5.

22. Kubota K, Lo E, Huttinot G, Andersen P, Maibach H. Plasma concentrations of betamethasone after topical application of betamethasone 17-valerate: comparison with oral administration. British Journal of Clinical Pharmacology. 1994;37(1):86-8.

23. Lithell H, Selinus I, Hosie J, Frithz G, Weiner L. Efficacy and safety of bisoprolol and atenolol in patients with mild to moderate hypertension: A double-blind, parallel group international multicentre study. European Heart Journal. 1987;8(suppl_M):55-64.

24. Bakheit AH, Ali R, Alshahrani AD, El-Azab AS. Chapter Two - Bisoprolol: A comprehensive profile. In: Al-Majed AA, editor. Profiles of Drug Substances, Excipients and Related Methodology. 46: Academic Press; 2021. p. 51-89.

25. Burke MJ, Ziegler DS, Bautista F, Attarbaschi A, Gore L, Locatelli F, et al. Phase 1b study of carfilzomib with induction chemotherapy in pediatric relapsed/refractory acute lymphoblastic leukemia. Pediatric Blood & Cancer. 2022;69(12):e29999.

26. food and Drug Administration (FDA). HIGHLIGHTS OF PRESCRIBING INFORMATION - KYPROLIS® (carfilzomib). 2020.

27. Brown J, Plummer R, Bauer TM, Anthony S, Sarantopoulos J, De Vos F, et al. Pharmacokinetics of carfilzomib in patients with advanced malignancies and varying degrees of hepatic impairment: an open-label, single-arm, phase 1 study. Exp Hematol Oncol. 2017;6:27.

28. Arnold CJ, Ericson J, Cho N, Tian J, Wilson S, Chu VH, et al. Cefepime and Ceftazidime Safety in Hospitalized Infants. Pediatr Infect Dis J. 2015;34(9):964-8.

29. Bristol-Myers Squibb Canada. PRODUCT MONOGRAPH - Maxipime 2008.

30. Mulhall A, de Louvois J, James J. Pharmacokinetics and safety of ceftriaxone in the neonate. Eur J Pediatr. 1985;144(4):379-82.

31. Zeng L, Wang C, Jiang M, Chen K, Zhong H, Chen Z, et al. Safety of ceftriaxone in paediatrics: a systematic review. Arch Dis Child. 2020;105(10):981-5.

32. Lee S, Kim SK, Lee DY, Chae SY, Byun Y. Pharmacokinetics of a new, orally available ceftriaxone formulation in physical complexation with a cationic analogue of bile acid in rats. Antimicrob Agents Chemother. 2006;50(5):1869-71.

33. Zalcman J, Pasternak Y, Kenan D, Dotan M, Gueta I, Kadmon G, et al. Safety of Cidofovir Treatment for Suspected or Confirmed Adenovirus Infection in Immunocompetent Pediatric Population. Pediatr Infect Dis J. 2024;43(3):198-202.

34. Bhadri VA, Lee-Horn L, Shaw PJ. Safety of Cidofovir in High-Risk Paediatric Patients. Blood. 2006;108(11):5296-.

35. Cundy KC. Clinical pharmacokinetics of the antiviral nucleotide analogues cidofovir and adefovir. Clin Pharmacokinet. 1999;36(2):127-43.

36. Gauda EB, Chavez-Valdez R, Northington FJ, Lee CKK, Rudek MA, Guglieri-Lopez B, et al. Clonidine for sedation in infants during therapeutic hypothermia with neonatal encephalopathy: pilot study. Journal of Perinatology. 2022;42(3):319-27.

37. Eberl S, Ahne G, Toni I, Standing J, Neubert A. Safety of clonidine used for long-term sedation in paediatric intensive care: A systematic review. British Journal of Clinical Pharmacology. 2021;87(3):785-805.

38. Basker S, Singh G, Jacob R. Clonidine In Paediatrics – A Review. Indian Journal of Anaesthesia. 2009;53(3):270-80.

39. Larsson P, Nordlinder A, Bergendahl HT, Lönnqvist PA, Eksborg S, Almenrader N, et al. Oral bioavailability of clonidine in children. Paediatr Anaesth. 2011;21(3):335-40.

40. Neffendorf JE, Mota PM, Xue K, Hildebrand GD. Efficacy and Safety of Phenylephrine 2.5% with Cyclopentolate 0.5% for Retinopathy of Prematurity Screening in 1246 Eye Examinations. European Journal of Ophthalmology. 2014;25(3):249-53.

41. Micromedex. 2024 [Available from: <https://www.micromedexsolutions.com/micromedex2/librarian/>.

42. SWISSPEDDOSE. 2024 [Available from: <https://db.swisspeddose.ch/search/>.

43. Agrahari V, Mandal A, Agrahari V, Trinh HM, Joseph M, Ray A, et al. A comprehensive insight on ocular pharmacokinetics. Drug Deliv Transl Res. 2016;6(6):735-54.

44. Lim D, Batilando M, Rajadurai V. Transient paralytic ileus following the use of cyclopentolate−phenylephrine eye drops during screening for retinopathy of prematurity. Journal of Paediatrics and Child Health. 2003;39(4):318-20.

45. Health Products Regulatory Authority. Summary of Product Characteristics - Minims Cyclopentolate Hydrochloride 1% w/v Eye Drops, solution. 2023.

46. Hebert AA. Desonide foam 0.05%: safety in children as young as 3 months. J Am Acad Dermatol. 2008;59(2):334-40.

47. Dhar S, Seth J, Parikh D. Systemic side-effects of topical corticosteroids. Indian J Dermatol. 2014;59(5):460-4.

48. Rutigliano G, Labout JJ. The bioavailability of orphenadrine hydrochloride after intramuscular and oral administration. J Int Med Res. 1982;10(6):447-50.

49. Peiró-Aventín B, Cabrera-Romero E, Mora-Ayestarán N, Domínguez F, González-López E, García-Pavía P. Safety and efficacy of diflunisal in transthyretin cardiac amyloidosis. Revista Española de Cardiología (English Edition). 2024;77(5):362-438.

50. Brogden RN, Heel RC, Pakes GE, Speight TM, Avery GS. Diflunisal: A Review of its Pharmacological Properties and Therapeutic Use in Pain and Musculoskeletal Strains and Sprains and Pain in Osteoarthritis. Drugs. 1980;19(2):84-106.

51. AA PHARMA INC. PRODUCT MONOGRAPH - DIFLUNISAL. 2014.

52. Bhayat SI, Gowda HM, Eisenhut M. Should dopamine be the first line inotrope in the treatment of neonatal hypotension? Review of the evidence. World J Clin Pediatr. 2016;5(2):212-22.

53. Wen L, Xu L. The efficacy of dopamine versus epinephrine for pediatric or neonatal septic shock: a meta-analysis of randomized controlled studies. Italian Journal of Pediatrics. 2020;46(1):6.

54. Driscoll DJ, Gillette PC, McNamara DG. The use of dopamine in children. The Journal of Pediatrics. 1978;92(2):309-14.

55. Murata K, Noda K, Kohno K, Samejima M. Bioavailability and pharmacokinetics of oral dopamine in dogs. J Pharm Sci. 1988;77(7):565-8.

56. Ceral J, Solar M. Doxazosin: safety and efficacy in the treatment of resistant arterial hypertension. Blood Pressure. 2009;18(1-2):74-7.

57. Elliott HL, Meredith PA, Reid JL. Pharmacokinetic overview of doxazosin. The American Journal of Cardiology. 1987;59(14):G78-G81.

58. Carr B, Giudice L, Dmowski WP, O'Brien C, Jiang P, Burke J, et al. Elagolix, an Oral GnRH Antagonist for Endometriosis-Associated Pain: A Randomized Controlled Study. J Endometr Pelvic Pain Disord. 2013;5(3):105-15.

59. Shebley M, Polepally AR, Nader A, Ng JW, Winzenborg I, Klein CE, et al. Clinical Pharmacology of Elagolix: An Oral Gonadotropin-Releasing Hormone Receptor Antagonist for Endometriosis. Clinical Pharmacokinetics. 2020;59(3):297-309.

60. Food and Drug Administration (FDA). NDA/BLA Multi‐Disciplinary Review and Evaluation - Elagolix Sodium. 2018.

61. TEVA Pharmaceutical Works Private Limited Company. SUMMARY OF PRODUCT CHARACTERISTICS - Entacapone Teva. 2021.

62. Poewe WH, Deuschl G, Gordin A, Kultalahti E-R, Leinonen M, Group tCS. Efficacy and safety of entacapone in Parkinson's disease patients with suboptimal Levodopa response: a 6-month randomized placebo-controlled double-blind study in Germany and Austria (Celomen study). Acta Neurologica Scandinavica. 2002;105(4):245-55.

63. McIntyre CM, Hanna BD, Rintoul N, Ramsey EZ. Safety of epoprostenol and treprostinil in children less than 12 months of age. Pulm Circ. 2013;3(4):862-9.

64. Degering J, Egenlauf B, Harutyunova S, Benjamin N, Salkić A, Xanthouli P, et al. Tolerability, safety and survival in patients with severe pulmonary arterial hypertension treated with intravenous epoprostenol (Veletri(®)): a prospective, 6-months, open label, observational, non-interventional study. Respir Res. 2023;24(1):18.

65. Nicolas LB, Krause A, Gutierrez MM, Dingemanse J. Integrated pharmacokinetics and pharmacodynamics of epoprostenol in healthy subjects. Br J Clin Pharmacol. 2012;74(6):978-89.

66. Witt O, Sait SF, Diez BD, Cardoso A, Reardon DA, Welsh L, et al. Efficacy and safety of erdafitinib in pediatric patients with advanced solid tumors and FGFR alterations in the phase 2 RAGNAR trial. Journal of Clinical Oncology. 2024;42(16_suppl):10002-.

67. De Zwart L, Snoeys J, Jacobs F, Li LY, Poggesi I, Verboven P, et al. Prediction of the drug-drug interaction potential of the α1-acid glycoprotein bound, CYP3A4/CYP2C9 metabolized oncology drug, erdafitinib. CPT Pharmacometrics Syst Pharmacol. 2021;10(9):1107-18.

68. Strawn JR, Moldauer L, Hahn RD, Wise A, Bertzos K, Eisenberg B, et al. A Multicenter Double-Blind, Placebo-Controlled Trial of Escitalopram in Children and Adolescents with Generalized Anxiety Disorder. J Child Adolesc Psychopharmacol. 2023;33(3):91-100.

69. Carandang C, Jabbal R, Macbride A, Elbe D. A review of escitalopram and citalopram in child and adolescent depression. J Can Acad Child Adolesc Psychiatry. 2011;20(4):315-24.

70. Food and Drug Administration (FDA). Lexapro® (escitalopram) Tablets - Highlights of prescribing information. 2019.

71. Inc. LC. CIPRALEX-Product monograph. 2023.

72. Galapagos NV. Jyseleca, INN-filgotinib - SUMMARY OF PRODUCT CHARACTERISTICS. 2024.

73. D'Amico F, Magro F, Peyrin-Biroulet L, Danese S. Positioning Filgotinib in the Treatment Algorithm of Moderate to Severe Ulcerative Colitis. J Crohns Colitis. 2022;16(5):835-44.

74. Tanaka Y, Genovese MC, Matsushima H. Long-Term Safety, Efficacy, and Patient-Centered Outcomes of Filgotinib in the Treatment of Rheumatoid Arthritis: Current Perspectives. Patient Prefer Adherence. 2023;17:2499-516.

75. Namour F, Anderson K, Nelson C, Tasset C. Filgotinib: A Clinical Pharmacology Review. Clin Pharmacokinet. 2022;61(6):819-32.

76. Luciani GB, Nichani S, Chang AC, Wells WJ, Newth CJ, Starnes VA. Continuous versus intermittent furosemide infusion in critically ill infants after open heart operations. Ann Thorac Surg. 1997;64(4):1133-9.

77. Jackson W, Taylor G, Selewski D, Smith PB, Tolleson-Rinehart S, Laughon MM. Association between furosemide in premature infants and sensorineural hearing loss and nephrocalcinosis: a systematic review. Matern Health Neonatol Perinatol. 2018;4:23.

78. Pacifici GM. Clinical pharmacology of furosemide in neonates: a review. Pharmaceuticals (Basel). 2013;6(9):1094-129.

79. Geller J, Kasahara M, Martinez M, Soresina A, Kashanian F, Endrikat J. Safety and Efficacy of Gadoxetate Disodium-Enhanced Liver MRI in Pediatric Patients Aged >2 Months to <18 Years-Results of a Retrospective, Multicenter Study. Magn Reson Insights. 2016;9:21-8.

80. Food and Drug Administration (FDA). HIGHLIGHTS OF PRESCRIBING INFORMATION - EOVIST (Gadoxetate Disodium). 2010.

81. Nguyen A, Miller WP, Gupta A, Lund TC, Schiferl D, Lam LSK, et al. Open-Label Pilot Study of Interferon Gamma-1b in Patients With Non-Infantile Osteopetrosis. JBMR Plus. 2022;6(3):e10597.

82. Roquilly A, Francois B, Huet O, Launey Y, Lasocki S, Weiss E, et al. Interferon gamma-1b for the prevention of hospital-acquired pneumonia in critically ill patients: a phase 2, placebo-controlled randomized clinical trial. Intensive Care Medicine. 2023;49(5):530-44.

83. Raghu G, Brown KK, Bradford WZ, Starko K, Noble PW, Schwartz DA, et al. A Placebo-Controlled Trial of Interferon Gamma-1b in Patients with Idiopathic Pulmonary Fibrosis. New England Journal of Medicine. 2004;350(2):125-33.

84. Food and Drug Administration (FDA). Actimmune (Interferon gamma 1-b). 2007.

85. Liebel-Flarsheim Company LLC. PRODUCT MONOGRAPH - CONRAY. 2022.

86. Prueksaritanont T, Lee MG, Hsu FH, Chiou WL. Absorption of iothalamate after oral administration and absorption enhancement by amino acids in dogs and rats. Biopharm Drug Dispos. 1986;7(5):463-78.

87. Blehaut H, Mircher C, Ravel A, Conte M, de Portzamparc V, Poret G, et al. Effect of leucovorin (folinic acid) on the developmental quotient of children with Down's syndrome (trisomy 21) and influence of thyroid status. PLoS One. 2010;5(1):e8394.

88. Lucchesi M, Guidi M, Fonte C, Farina S, Fiorini P, Favre C, et al. Pharmacokinetics of high-dose methotrexate in infants aged less than 12 months treated for aggressive brain tumors. Cancer Chemotherapy and Pharmacology. 2016;77(4):857-64.

89. Pfizer. Drug monograph - Leucovorin. 2016.

90. Laboratoire RIVA Inc. COMPLETE PRESCRIBING INFORMATION - RIVA LEUCOVORIN. 2019.

91. Espay AJ, Stocchi F, Pahwa R, Albanese A, Ellenbogen A, Ferreira JJ, et al. Safety and efficacy of continuous subcutaneous levodopa&#x2013;carbidopa infusion (ND0612) for Parkinson's disease with motor fluctuations (BouNDless): a phase 3, randomised, double-blind, double-dummy, multicentre trial. The Lancet Neurology. 2024;23(5):465-76.

92. Langrall HM, Joseph C. Evaluation of safety and efficacy of levodopa in Parkinson's disease and syndrome. Neurology. 1972;22(5_part_2):3-16.

93. APOTEX INC. PRODUCT MONOGRAPH - APO-LEVOCARB. 2022.

94. Robertson DR, Wood ND, Everest H, Monks K, Waller DG, Renwick AG, et al. The effect of age on the pharmacokinetics of levodopa administered alone and in the presence of carbidopa. Br J Clin Pharmacol. 1989;28(1):61-9.

95. Gouvernement du Canada. Summary Safety Review - Viscous Lidocaine 2% - Assessing the Potential Risk of Severe Side Effects in Infants and Young Children 2016 [Available from: <https://www.canada.ca/en/health-canada/services/drugs-health-products/medeffect-canada/safety-reviews/summary-safety-review-viscous-lidocaine-assessing-potential-risk-effects-infants-young-children.html>.

96. U.S. Food and Drug Administration (FDA). FDA Drug Safety Communication: FDA recommends not using lidocaine to treat teething pain and requires new Boxed Warning 2014 [Available from: <https://www.fda.gov/drugs/drug-safety-and-availability/fda-drug-safety-communication-fda-recommends-not-using-lidocaine-treat-teething-pain-and-requires#:~:text=In%202014%2C%20FDA%20reviewed%2022,or%20who%20had%20accidental%20ingestions>.

97. Rowbotham MC, Davies PS, Fields HL. Topical lidocaine gel relieves postherpetic neuralgia. Annals of Neurology. 1995;37(2):246-53.

98. Hoffman RJ. Viscous Lidocaine Treatment for Painful Oral Infections in Children: Disappointingly Dismissive of Pediatric Pain. Annals of Emergency Medicine. 2014;64(1):96-7.

99. Fishman M, Tirado C, Alam D, Gullo K, Clinch T, Gorodetzky CW. Safety and Efficacy of Lofexidine for Medically Managed Opioid Withdrawal: A Randomized Controlled Clinical Trial. J Addict Med. 2019;13(3):169-76.

100. Rehman SU, Maqsood MH, Bajwa H, Tameez Ud Din A, Malik MN. Clinical Efficacy and Safety Profile of Lofexidine Hydrochloride in Treating Opioid Withdrawal Symptoms: A Review of Literature. Cureus. 2019;11(6):e4827.

101. Food and Drug Administration (FDA). HIGHLIGHTS OF PRESCRIBING INFORMATION - LUCEMYRA™ (lofexidine) tablets. 2018.

102. Maniatis AK, Casella SJ, Nadgir UM, Hofman PL, Saenger P, Chertock ED, et al. Safety and Efficacy of Lonapegsomatropin in Children With Growth Hormone Deficiency: enliGHten Trial 2-Year Results. J Clin Endocrinol Metab. 2022;107(7):e2680-e9.

103. Ascendis Pharma A/S. SUMMARY OF PRODUCT CHARACTERISTIC - Lonapegsomatropin Ascendis Pharma. 2023.

104. Croft NM, Korczowski B, Kierkuś J, Caballero B, Thakur MK. Safety and efficacy of multimatrix mesalamine in paediatric patients with mild-to-moderate ulcerative colitis: a phase 3, randomised, double-blind study. eClinicalMedicine. 2023;65.

105. Berends SE, Strik AS, Löwenberg M, D’Haens GR, Mathôt RAA. Clinical Pharmacokinetic and Pharmacodynamic Considerations in the Treatment of Ulcerative Colitis. Clinical Pharmacokinetics. 2019;58(1):15-37.

106. Cadila Healthcare Ltd. Highlights of prescribing information - Mesalamine 2014.

107. Commander SJ, Gao J, Zinkhan EK, Heresi G, Courtney SE, Lavery AP, et al. Safety of Metronidazole in Late Pre-term and Term Infants with Complicated Intra-abdominal Infections. Pediatr Infect Dis J. 2020;39(9):e245-e8.

108. Desai A, Burdette S. Patient Safety and Efficacy of Metronidazole 1 g Intravenous Every 24 Hours. Open Forum Infect Dis. 2017;4(Suppl 1):S343.

109. Australia GoW. Metronidazole Monograph - Paediatric. In: Child and Adolescent Health Service, editor. 2021.

110. Feldt-Rasmussen U, Hughes D, Sunder-Plassmann G, Shankar S, Nedd K, Olivotto I, et al. Long-term efficacy and safety of migalastat treatment in Fabry disease: 30-month results from the open-label extension of the randomized, phase 3 ATTRACT study. Molecular Genetics and Metabolism. 2020;131(1):219-28.

111. Narita I, Ohashi T, Sakai N, Hamazaki T, Skuban N, Castelli JP, et al. Efficacy and safety of migalastat in a Japanese population: a subgroup analysis of the ATTRACT study. Clinical and Experimental Nephrology. 2020;24(2):157-66.

112. Almac Pharma Services (Ireland) Limited. SUMMARY OF PRODUCT CHARACTERISTICS - Galafold. 2023.

113. Hoffmann-La Roche Limited. OCREVUS® (ocrelizumab) - PRODUCT MONOGRAPH. 2023.

114. Hauser SL, Kappos L, Montalban X, Craveiro L, Chognot C, Hughes R, et al. Safety of Ocrelizumab in Patients With Relapsing and Primary Progressive Multiple Sclerosis. Neurology. 2021;97(16):e1546-e59.

115. Ovacik M, Lin K. Tutorial on Monoclonal Antibody Pharmacokinetics and Its Considerations in Early Development. Clin Transl Sci. 2018;11(6):540-52.

116. Kaguelidou F, Alberti C, Biran V, Bourdon O, Farnoux C, Zohar S, et al. Dose-Finding Study of Omeprazole on Gastric pH in Neonates with Gastro-Esophageal Acid Reflux Using a Bayesian Sequential Approach. PLOS ONE. 2016;11(12):e0166207.

117. Dipasquale V, Cicala G, Spina E, Romano C. A Narrative Review on Efficacy and Safety of Proton Pump Inhibitors in Children. Frontiers in Pharmacology. 2022;13.

118. Food and Drug Administration (FDA). HIGHLIGHTS OF PRESCRIBING INFORMATION - PRILOSEC. 2016.

119. Kearns GL, Andersson T, James LP, Gaedigk A, Kraynak RA, Abdel-Rahman SM, et al. Omeprazole Disposition in Children following Single-Dose Administration. The Journal of Clinical Pharmacology. 2003;43(8):840-8.

120. Abd-Elsalam S, El-Kalla F, Ali LA, Mosaad S, Alkhalawany W, Elemary B, et al. Pilot study of orphenadrine as a novel treatment for muscle cramps in patients with liver cirrhosis. United European Gastroenterol J. 2018;6(3):422-7.

121. Kalia S, Nath P, Pathak M, Anand AC. Treatment of Muscle Cramps in Patients With Cirrhosis of Liver: A Systematic Review. Journal of Clinical and Experimental Hepatology. 2022;12(3):980-92.

122. Shore SN, Britnell SR, Brown JN. Safety analysis of long-term phenazopyridine use for radiation cystitis. J Oncol Pharm Pract. 2020;26(2):306-11.

123. Erfa Canada Inc. PRODUCT MONOGRAPH - PYRIDIUM. 2010.

124. Eastham jH PP. Phenazopyridine: Treasure Island (FL): StatPearls Publishing; 2024 [Available from: <https://www.ncbi.nlm.nih.gov/books/NBK580545/>.

125. Ali Ibrahim AI, Mendoza B, Stanford FC, Malhotra S. Real-World Experience of the Efficacy and Safety of Phentermine Use in Adolescents: A Case Series. Childhood Obesity. 2023;19(8):535-40.

126. Lei XG, Ruan JQ, Lai C, Sun Z, Yang X. Efficacy and Safety of Phentermine/Topiramate in Adults with Overweight or Obesity: A Systematic Review and Meta-Analysis. Obesity (Silver Spring). 2021;29(6):985-94.

127. iNova Pharmaceuticals (Australia) Pty Limited. AUSTRALIAN PRODUCT INFORMATION – METERMINE

(PHENTERMINE) CAPSULE. 2021.

128. Wu YE, Hou SS, Fang ZY, Tang BH, Yao BF, Dong YN, et al. Clinical utiliy of a model-based piperacillin dose in neonates with early-onset sepsis. Br J Clin Pharmacol. 2022;88(3):1179-88.

129. Bush K. CHAPTER 15 - Other β-lactam antibiotics. In: Finch RG, Greenwood D, Norrby SR, Whitley RJ, editors. Antibiotic and Chemotherapy (Ninth Edition). London: W.B. Saunders; 2010. p. 226-44.

130. Wolf MF, Simon A. The use of piperacillin–tazobactam in neonatal and paediatric patients. Expert Opinion on Drug Metabolism & Toxicology. 2009;5(1):57-69.

131. Lau Moon Lin M, Robinson PD, Flank J, Sung L, Dupuis LL. The Safety of Prochlorperazine in Children: A Systematic Review and Meta-Analysis. Drug Saf. 2016;39(6):509-16.

132. Isah A, Rawlins M, Bateman D. Clinical pharmacology of prochlorperazine in healthy young males. British Journal of Clinical Pharmacology. 1991;32(6):677-84.

133. Kamp CB, Petersen JJ, Faltermeier P, Juul S, Siddiqui F, Barbateskovic M, et al. Beneficial and harmful effects of tricyclic antidepressants for adults with major depressive disorder: a systematic review with meta-analysis and trial sequential analysis. BMJ Ment Health. 2024;27(1).

134. Rief W, Nestoriuc Y, von Lilienfeld-Toal A, Dogan I, Schreiber F, Hofmann SG, et al. Differences in Adverse Effect Reporting in Placebo Groups in SSRI and Tricyclic Antidepressant Trials. Drug Safety. 2009;32(11):1041-56.

135. Roxane Laboratories I. Protriptyline Hydrochloride Tablets, USP. 2014.

136. Geoerger B, Bourdeaut F, DuBois SG, Fischer M, Geller JI, Gottardo NG, et al. A Phase I Study of the CDK4/6 Inhibitor Ribociclib (LEE011) in Pediatric Patients with Malignant Rhabdoid Tumors, Neuroblastoma, and Other Solid Tumors. Clinical Cancer Research. 2017;23(10):2433-41.

137. Bardia A, Modi S, Gregor MC-M, Kittaneh M, Marino AJ, Matano A, et al. Phase Ib/II study of LEE011, everolimus, and exemestane in postmenopausal women with ER+/HER2-metastatic breast cancer. Journal of Clinical Oncology. 2014;32(15_suppl):535-.

138. Ji Y, Abdelhady AM, Samant TS, Yang S, Rodriguez Lorenc K. Evaluation of Absolute Oral Bioavailability and Bioequivalence of Ribociclib, a Cyclin-Dependent Kinase 4/6 Inhibitor, in Healthy Subjects. Clin Pharmacol Drug Dev. 2020;9(7):855-66.

139. Dong Y, Yue M, Hu M. The Efficacy and Safety of Different Dosages of Rituximab for Adults with Immune Thrombocytopenia: A Systematic Review and Meta-Analysis. Biomed Res Int. 2021;2021:9992086.

140. Merkel PA, Niles JL, Mertz LE, Lehane PB, Pordeli P, Erblang F. Long-Term Safety of Rituximab in Granulomatosis With Polyangiitis and in Microscopic Polyangiitis. Arthritis Care Res (Hoboken). 2021;73(9):1372-8.

141. Brogan P, Yeung RSM, Cleary G, Rangaraj S, Kasapcopur O, Hersh AO, et al. Phase IIa Global Study Evaluating Rituximab for the Treatment of Pediatric Patients With Granulomatosis With Polyangiitis or Microscopic Polyangiitis. Arthritis Rheumatol. 2022;74(1):124-33.

142. Food and Drug Administration (FDA). HIGHLIGHTS OF PRESCRIBING INFORMATION - SYLVANT. 2018.

143. van Rhee F, Casper C, Voorhees PM, Fayad LE, van de Velde H, Vermeulen J, et al. A phase 2, open-label, multicenter study of the long-term safety of siltuximab (an anti-interleukin-6 monoclonal antibody) in patients with multicentric Castleman disease. Oncotarget. 2015;6(30):30408-19.

144. van Rhee F, Casper C, Voorhees PM, Fayad LE, Gibson D, Kanhai K, et al. Long-term safety of siltuximab in patients with idiopathic multicentric Castleman disease: a prespecified, open-label, extension analysis of two trials. The Lancet Haematology. 2020;7(3):e209-e17.

145. Fayed SB, Sutton AM, Turner TL, McAllister TA. The prophylactic use of ticarcillin/clavulanate in the neonate. Journal of Antimicrobial Chemotherapy. 1987;19(1):113-8.

146. (FDA) FaDA. HIGHLIGHTS OF PRESCRIBING INFORMATION - Timentin. 2014.

147. Sanders CV, Marier RL, Aldridge KE, Derks FW, Martin DH. Safety and effectiveness of ticarcillin plus clavulanic acid in the treatment of community-acquired acute pyelonephritis in adult women. Am J Med. 1985;79(5b):96-100.

148. Injury LCaRIoD-IL. Ticarcillin-Clavulanate: Bethesda (MD): National Institute of Diabetes and Digestive and Kidney Diseases; 2012 [Available from: <https://www.ncbi.nlm.nih.gov/books/NBK548183/>.

149. Gian Maria Pacifici. Clinical pharmacology of tobramycin in infants and children Clinical and Medical Investigations 2020;5: 3-11.

150. Geller DE, Pitlick WH, Nardella PA, Tracewell WG, Ramsey BW. Pharmacokinetics and bioavailability of aerosolized tobramycin in cystic fibrosis. Chest. 2002;122(1):219-26.

151. Banerjee SK, Jagannath C, Hunter RL, Dasgupta A. Bioavailability of tobramycin after oral delivery in FVB mice using CRL-1605 copolymer, an inhibitor of P-glycoprotein. Life Sci. 2000;67(16):2011-6.

152. Armstrong DK, Spriggs D, Levin J, Poulin R, Lane S. Hematologic Safety and Tolerability of Topotecan in Recurrent Ovarian Cancer and Small Cell Lung Cancer: An Integrated Analysis. The Oncologist. 2005;10(9):686-94.

153. Abudou M, Zhong D, Wu T, Wu X. Topotecan for ovarian cancer. Cochrane Database of Systematic Reviews. 2008(2).

154. Limited HU. SUMMARY OF PRODUCT CHARACTERISTICS - Topotecan. 2022.

155. Inc. AH. PRODUCT MONOGRAPH - Topotecan hydrochloride for Injection. 2019.

156. Kubeyinje E. Evaluation of the efficacy and safety of 0.05% tretinoin cream in the treatment of plane warts in Arab children. Journal of Dermatological Treatment. 1996;7(1):21-2.

157. Berger R, Barba A, Fleischer A, Leyden JJ, Lucky A, Pariser D, et al. A double-blinded, randomized, vehicle-controlled, multicenter, parallel-group study to assess the safety and efficacy of tretinoin gel microsphere 0.04% in the treatment of acne vulgaris in adults. Cutis. 2007;80(2):152-7.

158. Kang S, Bergfeld W, Gottlieb AB, Hickman J, Humeniuk J, Kempers S, et al. Long-term efficacy and safety of tretinoin emollient cream 0.05% in the treatment of photodamaged facial skin: a two-year, randomized, placebo-controlled trial. Am J Clin Dermatol. 2005;6(4):245-53.

159. Food and Drug Administration (FDA). HIGHLIGHTS OF PRESCRIBING INFORMATION - VESANOID. 2023.

160. Bausch Health CI. PRODUCT MONOGRAPH - RETIN-A. 2022.

161. Limited GI. SUMMARY OF PRODUCT CHARACTERISTICS - Incruse Ellipta. 2024.

162. Feldman G, Walker RR, Brooks J, Mehta R, Crater G. 28-Day safety and tolerability of umeclidinium in combination with vilanterol in COPD: A randomized placebo-controlled trial. Pulmonary Pharmacology & Therapeutics. 2012;25(6):465-71.

163. Donohue JF, Maleki-Yazdi MR, Kilbride S, Mehta R, Kalberg C, Church A. Efficacy and safety of once-daily umeclidinium/vilanterol 62.5/25 mcg in COPD. Respir Med. 2013;107(10):1538-46.

164. Food and Drug Administration (FDA). CLINICAL PHARMACOLOGY AND BIOPHARMACEUTICS REVIEW(S) - INCRUSE ELLIPTA. 2013.
